# The Epithelial αvβ3-Integrin Boosts the MYD88-Dependent TLR2 Signaling in Response to Viral and Bacterial Components

**DOI:** 10.1371/journal.ppat.1004477

**Published:** 2014-11-06

**Authors:** Tatiana Gianni, Gabriella Campadelli-Fiume

**Affiliations:** Department of Experimental, Diagnostic and Specialty Medicine, Alma Mater Studiorum–University of Bologna, Bologna, Italy; University of California, Irvine, United States of America

## Abstract

TLR2 is a cell surface receptor which elicits an immediate response to a wide repertoire of bacteria and viruses. Its response is usually thought to be proinflammatory rather than an antiviral. In monocytic cells TLR2 cooperates with coreceptors, e.g. CD14, CD36 and αMβ2-integrin. In an earlier work we showed that αvβ3-integrin acts in concert with TLR2 to elicit an innate response to HSV, and to lipopolysaccharide. This response is characterized by production of IFN-α and -β, a specific set of cytokines, and NF-κB activation. We investigated the basis of the cooperation between αvβ3-integrin and TLR2. We report that β3-integrin participates by signaling through Y residues located in the C-tail, known to be involved in signaling activity. αvβ3-integrin boosts the MYD88-dependent TLR2 signaling and IRAK4 phosphorylation in 293T and in epithelial, keratinocytic and neuronal cell lines. The replication of ICP0minus HSV is greatly enhanced by DN versions of MYD88, of Akt – a hub of this pathway, or by β3integrin-silencing. αvβ3-integrin enables the recruitment of TLR2, MAL, MYD88 at lipid rafts, the platforms from where the signaling starts. The PAMP of the HSV-induced innate response is the gH/gL virion glycoprotein, which interacts with αvβ3-integrin and TLR2 independently one of the other, and cross-links the two receptors. Given the preferential distribution of αvβ3-integrin to epithelial cells, we propose that αvβ3-integrin serves as coreceptor of TLR2 in these cells. The results open the possibility that TLR2 makes use of coreceptors in a variety of cells to broaden its spectrum of activity and tissue specificity.

## Author Summary

In an earlier work we showed that a relevant contribution to the overall IFN-based antiviral response of the cell to herpes simplex virus is exerted by αvβ3-integrin which acts in concert with TLR2 in eliciting this response. Major characteristics of this branch of the innate response are the secretion of IFN-α and -β, of a specific set of cytokines, and the activation of NF-κB. The response is elicited also by LPS, indicating that the αvβ3-integrin TLR2 sentinels sense both bacteria and viruses. The IFN response is usually thought to be elicited by the endosomal and cytoplasmic sensors. Here we have investigated the basis of the αvβ3-integrin–TLR2 response, and found that αvβ3-integrin acts through its signaling C-tail, and boosts the MYD88- IRAK4-dependent TLR2 response. This is seen also in epithelial and neuronal cells which exemplify targets of HSV infection. Altogether, the results argue that αvβ3-integrin may serve as a coreceptor of TLR2 in epithelial cells. A point of novelty is that the TLR2 coreceptors known to date - CD14, CD36 and αMβ2-integrins - are typical of monocytic-derived cells (macrophages, DCs). To our knowledge a TLR2 coreceptor for epithelial cells was not known to date.

## Introduction

The toll like receptors (TLRs) constitute a major defensive system of the cell against invasion from bacteria and viruses, and endogenous DAMPs (danger associated molecular patterns) [1]. Some of them, including TLR2 and 4, are present at the cell surface and mount the immediate branch of the innate response, before the invading microorganism or its components are internalized into the cell, and before the cytoplasmic sensors come into play. Initially described as an antibacterial sentinel [2], TLR2 emerged also as an antiviral sentinel [3], [4], and, indeed, it is characterized by the wide spectrum of bacteria, viruses and DAMPs which it senses [5]. The general view is that TLR2 favours a proinflammatory response.

In the past few years αvβ3-integrin and TLR2 were shown to act in concert to elicit a response to lipopeptide, to lipopolysaccharide (LPS) and to herpes simplex virus (HSV), a large DNA virus [6]–[9]. Specifically, our laboratory reported that in cells positive for both αvβ3-integrin and TLR2, IFN (interferon) α and β, and a specific set of cytokines - IL (interleukin) 2 and IL10 - were highly upregulated, and NF-κB was activated in response to HSV infection or exposure to a commercial source of LPS. By contrast, in cells negative for TLR2, the IFN-α and -β production and the NF-κB response were very low. In loss of function experiments, the silencing of β3-integrin in TLR2-positive cells dramatically reduced the IFN-α and-β production and the NF-κB response. The β3-integrin-silencing in TLR2-negative cells practically abolished the IFN and NF-κB response [7]–[9]. Importantly, the activation of IFN-α and -β and of NF-κB was detected not only in the model 293T cells, but also in epithelial, keratinocytic and neuronal cell lines, i.e. in cells which are models of the cells targeted by HSV *in vivo* [7]. These findings highlight that the branch of the innate response dependent on the αvβ3-integrin and TLR2 axis makes a significant contribution to the overall IFN production in HSV-infected cells. They contrast with the prevalent view that the IFN antiviral effect is triggered mainly by the endosomal and the cytoplasmic sensors [10]–[13].

The herpes simplex virion component which elicits this immediate response is the envelope glycoprotein gH, which forms a heterodimer with gL [9]. gH/gL are part of the fusion machinery in HSV required for HSV entry into the cell [14], [15]. They interact physically with αvβ3-integrin at low affinity [8]. They also interact with TLR2 by coimmunoprecipitation in αvβ3-integrin positive cells [9]. In turn, αvβ3-integrin and TLR2 interact with each other in a ligand-independent manner, i.e. in resting conditions [6], [8]. HSV evades this immediate response as soon as it enters the cell, at the onset of viral protein synthesis, by aid of the immediate early protein ICP0 (infected cell protein 0) [8], [16]. Importantly, the same effects were elicited by HSV and by a commercial preparation of LPS, hence the repertoire of microbial components capable to elicit this branch of the innate response is broad [7], [8].

The objective of this work was to shed light on the mechanisms by which αvβ3-integrin and TLR2 act in concert to elicit this branch of the innate response to HSV and LPS. At large, two scenarios were envisioned. In the first, the two receptors – αvβ3-integrin and TLR2 – increase the efficiency of virus entry into cell or of LPS binding-uptake into the cell. The alternative scenario envisions that, since each of the two receptors exhibits intrinsic signaling activity, the concerted response results from boosting of one or the other signaling activity. Against the first possibility argues the finding that the extent of HSV infection was essentially similar in wt- and in β3-integrin-silenced cells [8]. To preliminarily verify the second possibility we examined if αvβ3-integrin participates in this response through critical Y residues of its cytoplasmic tail, known to be involved in signaling. Subsequently, we examined the intermediates in the pathways downstream of TLR2 and of αvβ3-integrin and found that αvβ3-integrin boosts the recruitment of MYD88 (myeloid differentiation primary response gene 88) to TLR2 and the phosphorylation of IRAK4 (Interleukin-1 receptor-associated kinase). The reverse effect, boosting of αvβ3-integrin signaling by TLR2, was not detected. Thus, the concerted αvβ3-integrin and TLR2 response rests on augmentation by αvβ3-integrin of the MYD88-dependent TLR2 signaling.

## Results

### The NF-κB activation induced by HSV, or LPS, involves signaling through the cytoplasmic tail of β3-integrin

**Figure 1:**
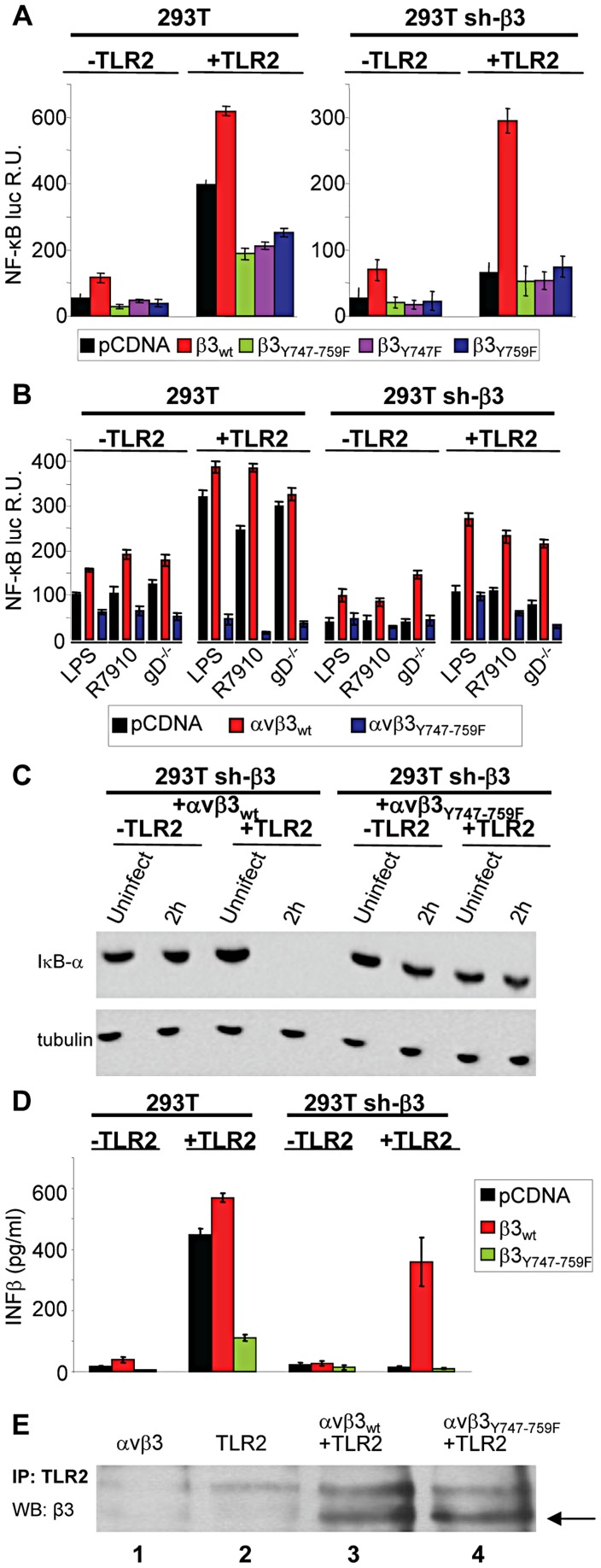
The NF-κB activation induced by HSV or LPS involves signaling through the cytoplasmic tail of β3-integrin. (A and B) Wt 293T (293T) or β3-integrin silenced (sh-β3) 293T cells were transfected with plasmids encoding TLR2, or empty plasmid, NF-κB-luc and Renilla luciferase, and one of the following β3-integrin forms, β3_wt_, β3_Y747F_, β3_Y759F_ or β3_Y747-759F_, along with αv-integrin subunit Starting at 12 h after transfection, cells were cultured in pre-exhausted medium [8] for 48–72 h, and then infected with the ΔICP0 recombinant R7910 (20 PFU/cell), or the gD deletion HSV (gD^−/−^) for 6 h, or exposed to lipopolysaccharide (LPS) (100 ng/ml) for 4 h. NF-κB-luc was measured as detailed [8], and expressed as relative units (RU) of luciferase∶Renilla ratio. Each value represents the average of triplicate samples ± SD. (C) β3-integrin silenced (sh-β3) 293T were transfected with wt-β3-integrin or β3_Y747-759F_, plus αv-integrin and TLR2, where indicated. Cells were cultured in pre-exhausted medium [8] for 48–72 h, starting at12 after transfection, and then infected with R7910 (20 PFU/cell) and harvested 2 after infection, or uninfected. Total cell lysates were subjected to SDS-PAGE (sodium dodecyl sulphate polyacrylamide gel electrophoresis) and blotted for IκB-α, or tubulin as loading control. (D) Wt 293T (293T) or sh-β3 293T cells were transfected with plasmids encoding wt-β3-integrin or β3_Y747-759F_, and TLR2 as indicated. After culturing in pre-exhausted medium for 48–72 h, cells were infected with R7910 (20 PFU/cell). IFN-β was quantified in the culture medium harvested 48 h after infection, by means of VeriKin kit. Each value represents the average of triplicate samples ± SD. (E) Both αvβ3_wt_ and αvβ3_Y747-759F_ interact with TLR2 by coimmunoprecipitation. Sh-β3 293T cells were transfected with plasmids encoding αv+β3_wt_integrin (lane 1), TLR2-Flag (lane 2), TLR2-Flag plus αv+β3_wt_integrin (lane 3), or TLR2-Flag plus αv+β3_Y747-759F_ (lane 4). TLR2-Flag was immunoprecipitated with anti-Flag MAb (IP: TLR2). Complexes were harvested by means of protein G-Sepharose. Co-immunoprecipitated proteins were separated by PAGE. The β3-integrins (marked by arrow) were detected by WB with PAb to β3-integrin (WB: β3).

As a first line of evidence that the response to HSV, or to LPS, mediated in concert by αvβ3-integrin and TLR2 results from a signaling activity, we asked whether integrin participates through its cytoplasmic tail, namely through residues Y747 and Y759 known to undergo phosphorylation upon integrin activation. To avoid the interference from endogenous β3-integrin, we employed 293T cells in which β3-integrin was stably silenced (named 293T sh-β3 cells) [8], and transfected them with plasmid encoding the wt- or mutant forms of β3-integrin carrying the single Y747F, or Y759F substitution, or both (β3-integrin_Y747_, β3-integrin_Y759_, β3-integrin_Y747-Y759_) [17]. 293T cells fail to express TLR2, hence cells were transfected, or not, with TLR2, as indicated. Comparison was with non-silenced 293T cells, expressing or not TLR2, plus the indicated β3-integrin isoform. To measure the NF-κB response, cells were additionally transfected with a plasmid encoding firefly luciferase under NF-κB regulated promoter (pNF-κB-luc). Renilla luciferase was included to account for variations in transfection efficiency. For virus-induced stimulation, we made use of the HSV mutant named R7910, which carries the deletion of the gene encoding the immediate early protein ICP0 [18]. The immediate innate response to HSV is counteracted by this viral protein, and is therefore best detected in its absence [8]. Previously, we ascertained that HSV-1 virions entered β3-integrin-positive or -negative cells with similar efficiency. This result ruled out that the lower innate response seen in sh-β3 cells could be attributed to lower amount of virus infecting the cells [8]. The results in Fig. 1 A, right panel, shows that, in the presence of TLR2, R7910 elicited NF-κB activation in sh-β3 cells expressing wt β3-integrin, but not any of the integrin mutants. For comparison, Fig. 1A, left panel, shows that R7910 elicited NF-κB activation in non silenced cells overexpressing wt β3-integrin, in agreement with previous data [8]. The activation was reduced by about 60% in cells overexpressing the integrin mutants (note the different scale in Fig. 1A, right and left panels). As reported previously, the NF-κB activation is high in the presence but not in the absence of TLR2, reflecting the αvβ3-integrin–TLR2 concerted action.

In the next series of experiments, and in some of the subsequent experiments, we made use of gD^−/−^ virions rather than of R7910. gD is one of the essential glycoprotein for HSV entry into the cell [14], [15]. gD^−/−^ virions carry the deletion of the gD gene, lack gD in the virion envelope, are competent for attachment to but not for entry into cells. Because they elicit an innate response - albeit at levels somewhat lower than the gD-containing virions - they enabled us to rule out that any effect on the innate response seen with the β3-integrin mutant might be attributable to a reduction in virus entry. The response to a commercial LPS preparation able to elicit a TLR2 response [8] was assayed in parallel. Fig. 1 B shows that the substitution of wt-β3-integrin with the β3-integrin_Y747-Y759_ grossly reduced the NF-κB activation elicited by gD^−/−^ virions, or LPS; this occurred both in sh-β3 (right panel) and in wt cells (left panel).

Changes to endogenous NF-κB were measured through the degradation of IκB-α, an cytoplasmic inhibitory component of NF-κB pathway, which undergoes degradation when NF-κB is activated and then translocated to the nucleus. Fig. 1 C shows that in sh-β3 cells which express the β3-integrin_Y747-Y759_ mutant and TLR2, and do not enable NF-κB activation, IκB-α was not degraded. By contrast, in sh-β3 which express the wt β3-integrin and TLR2, IκB-α was completely degraded 2 h after exposure of cells to R7910. Previous studies showed that the production of IFN-α and -β, and of the specific cytokines followed the same pattern as NF-κB activation [7], [8]. We ascertained whether the mutant form β3-integrin_Y747-Y759_ hampered IFN-β response. sh-β3 or wt 293 cells, positive or negative for TLR2, expressing or wt or mutant β3-integrin were infected with R7910. The culture medium was harvested at 48 h after infection, and the secretion of IFN-β was measured by ELISA. Fig. 1 D shows that IFN-β was secreted only by cells expressing wt, but not the mutant β3-integrin_Y747-Y759_, in agreement with the NF-κB response.

Gerold *et al* and our laboratory [6], [8] showed that αvβ3-integrin and TLR2 interact in a ligand-independent manner, as seen by co-immunoprecipitation. We verified that the β3-integrin_Y747-Y759_ mutant maintains the ability to interact with TLR2. TLR2-Flag was immunoprecipitated from β3-integrin–silenced cells, transfected with wt-β3-integrin or β3-integrin_Y747-Y759_, plus TLR2-Flag. Fig. 1 E shows that β3-integrin was coimmunoprecipitated by TLR2-Flag, irrespective of mutations in the cytoplasmic tail (compare lanes 3 and 4). The results indicate that the innate response to HSV, or to LPS, dependent on the concerted αvβ3-integrin–TLR2 action is hampered when a mutant form of β3-integrin defective in phosphorylation replaces wt β3-integrin. Cumulatively, they demonstrate that the immediate innate response triggered by the integrin–TLR2 axis results from a signaling activity.

### The recruitment of MYD88 to TLR2 and the IRAK4 phosphorylation are decreased in β3-integrin-silenced cells

**Figure 2:**
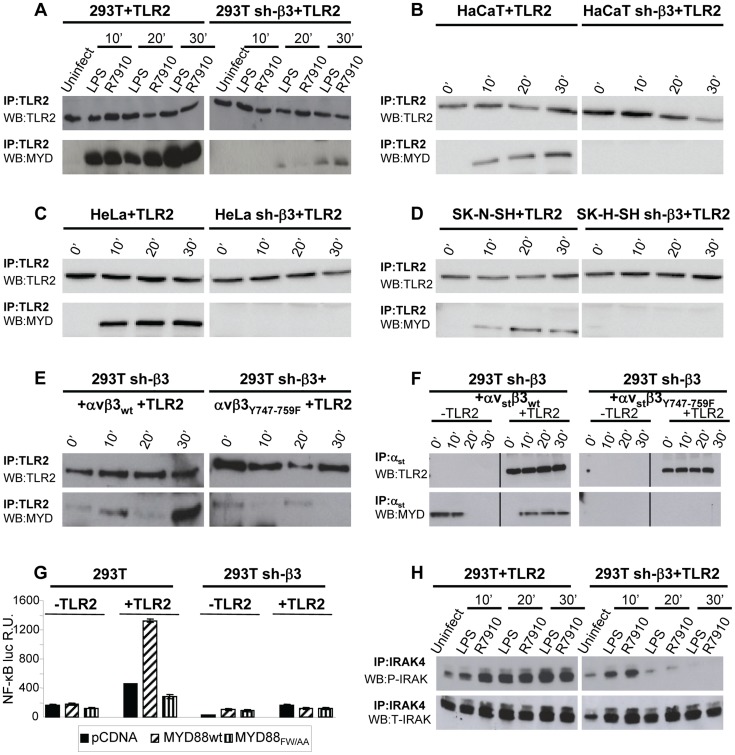
The recruitment of MYD88 to TLR2 and IRAK-4 phosphorylation are decreased in β3-integrin–silenced cells. (A–D) Recruitment of MYD88 to TLR2 in wt and β3-integrin–silenced sh-β3 293T (A), HaCaT (B), HeLa (C), or SK-N-SH cells, detected by coimmunoprecipitation. Wt or β3-integrin silenced cells were transfected with TLR2-Flag and MYD88-HA. Cells were exposed to R7910 (60 PFU/cell) or LPS (300 ng/ml) for 10, 20 and 30 min. TLR2-Flag was immunoprecipitated with anti-Flag MAb (IP: TLR2). The proteins retained by Protein G-Sepharose were separated by PAGE and revealed by WB with MAb to HA (WB: MYD) or to Flag (WB: TLR2). (E) The recruitment of MYD88 is inhibited when wt β3-integrin is substituted with the β3_Y747-759F_. sh-β3 cells were transfected with plasmids encoding TLR2-Flag, MYD88-HA, αv-integrin, β3_wt_-integrin, or β3_Y747-759F_-integrin. Exposure of cells to R7910, immunoprecipitation and WB analysis were as detailed in Fig. 2A. (F) MYD88 is recruited to a complex with integrin, in the absence of TLR2. 293T sh-β3 cells were transfected with plasmids encoding strep-labeled αv-integrin (αv_st_), β3_wt_ or β3_Y747-759F_-integrin, MYD88-HA, plus or minus TLR2-Flag, as indicated. Cells were exposed to R7910 for 10, 20, 30 min. αv_st_ was harvested by means of Strep-Tactin Sepharose. The retained proteins (IP: αv_st_) were separated by PAGE and revealed by WB to Flag (WB: TLR2), or to HA (WB: MYD). (G) MYD88 is required for the NF-κB activation dependent on αvβ3-integrin and TLR2. 293T or sh-β3 293T cells were transfected with plasmids encoding TLR2 (+TLR2), or empty plasmid (-TLR2), NF-κB-luc and Renilla luciferase, MYD88-HA (MYD88wt) or dominant negative version MYD88_FW/AA_. NF-κB-luc was quantified as described [8]. Each value represents the average of triplicate samples ± SD. (H) 293T or 293T sh-β3 cells were transfected with TLR2-Flag and MYD88-HA. Cells were exposed to R7910 or LPS as detailed in panel A. Endogenous IRAK-4 was immunoprecipitated with PAb to IRAK4 (IP: IRAK4). Phosphorylated IRAK4 (P-IRAK) and total IRAK (T-IRAK) were detected by means of anti phospho-IRAK4 PAb or anti-IRAK4 PAb.

To shed light on the mechanism by which αvβ3-integrin and TLR2 act in concert, we asked whether integrin boosts the TLR2 signaling response, or *viceversa*, whether TLR2 boosts the αvβ3-integrin signaling. To address the first question, we analyzed the typical intermediates downstream of TLR2 and asked whether their recruitment/activation was higher in integrin-positive (wt) than in β3-integrin-silenced cells. TLR2 signals through the recruitment of MYD88, followed by phosphorylation of IRAK 1 and 4 [19]. TLR2-Flag and hemagglutin (HA)-tagged MYD88 (MYD88-HA) were expressed in 293T or sh-β3 cells. Cells were exposed to R7910 (60 PFU/cell) or to LPS (300 ng/ml) for 10, 20, 30 min. Fig. 2 A shows that the amount of MYD88-HA coimmunoprecipitated by TLR2-Flag was dramatically decreased in sh-β3 cells (right panels), as compared to non-silenced cells (left panels). The recruitment of MYD88 to TLR2 in response to LPS was also dramatically decreased in sh-β3 cells (Fig. 2 A). The defective recruitment of MYD88 to TLR2 upon silencing of β3-integrin was seen also in cells other than 293T cells. We selected the keratinocytic HaCaT, the epithelial HeLa and the neuronal SK-N-SH cell lines, which are models of the cells targeted by HSV *in vivo*. Previous studies showed that silencing of β3-integrin in the above cell lines dramatically reduced both IFN-β production and NF-κB activation [7]. The indicated cells were transfected as indicated for panel A. Fig. 2 B–D show that also in HaCaT, HeLa and SK-N-SH cells, the silencing of β3-integrin resulted in strong impairment of MYD88 coimmunoprecipitated by TLR2, upon exposure of cells to R7910.

Next, we investigated whether the expression of the β3-integrin_Y747-Y759_ mutant, defective in signaling, and defective in the HSV- or LPS-induced activation of NF-κB, resulted in a reduced recruitment of MYD88 to TLR2. sh-β3 cells, transfected with wt- or β3-integrin_Y747-Y759_ were additionally transfected with TLR2-Flag and MYD88-HA. Fig. 2 E shows that the MYD88 recruitment to TLR2 was strongly reduced in cells expressing the β3-integrin mutant (right panel).

We then asked whether MYD88 can be recruited to a complex with integrin in the absence of TLR2, and whether this complex formation is hampered when the wt β3-integrin is substituted with the β3-integrin_Y747-Y759_ mutant. Sh-β3 cells were transfected with a form of αv-integrin carrying the double-strep epitope (named αv_strep_ or αv_st_), wt-β3-integrin or β3-integrin_Y747-Y759_, plus or minus TLR2. The transfected cells were exposed to R7910 for 10, 20, or 30 min. αv_st_-integrin was harvested by means of Strep-Tactin Sepharose. The co-precipitations in Fig. 2 F show that MYD88 was co-precipitated by αv_strep_wt-β3-integrin (left panel), but not by αv_strep_β3-integrin_Y747-Y759_ mutant (right panel), irrespective of the presence or absence of TLR2. Whether this results from a direct MYD88–αvβ3-integrin interaction, or from an indirect interaction remains to be investigated.

To provide evidence for a functional role of MYD88 in the αvβ3-integrin–TLR2 signaling pathway, we made use of a DN (dominant negative) version of MYD88, named MYD88_FW/AA_ [20]. MYD88_FW/AA_ carries the indicated substitutions in α helix E (αE). It can be recruited to TLR2 but is defective in downstream signaling [20]. 293T or sh-β3 cells, plus or minus TLR2, were transfected with MYD88_FW/AA_ or wt-MYD88, plus NF-κB-luc and Renilla luciferase plasmids. The transfected cells were infected with R7910 for 6 h. Fig. 2 G shows that NF-κB activation was dramatically decreased in cells expressing the DN MYD88_FW/AA_.

Downstream of MYD88, the TLR2 signaling cascade involves the phosphorylation of IRAK1, and of IRAK4. We compared the extent of IRAK4 phosphorylation in 293T and in sh-β3 cells, transfected with MYD88 and TLR2. Cells were infected with R7910, or exposed to LPS. Endogenous IRAK4 was immunoprecipitated. The immunoblot for phospho-IRAK4 (P-IRAK4) shows a dramatic decrease of the phosphorylated form in β3-integrin–silenced cells, while the total amount (T-IRAK4) was not substantially modified (Fig. 2 H).

Cumulatively, the results show that the absence of αvβ3-integrin, or a C-tail mutant form of β3-integrin, greatly decreased the amount of MYD88 recruited to TLR2 in a number of cell lines which are models of cells targeted by HSV *in vivo*. The results further show that the amount of P-IRAK4, and the HSV-induced NF-κB activation strongly depend on a signaling-competent form of MYD88. We conclude that the αvβ3-integrin–TLR2 concerted action entails a strong boost of the MYD88-dependent TLR2 signaling by αvβ3-integrin.

### HSV induces Src phosphorylation dependent on β3-integrin and independent of TLR2

**Figure 3:**
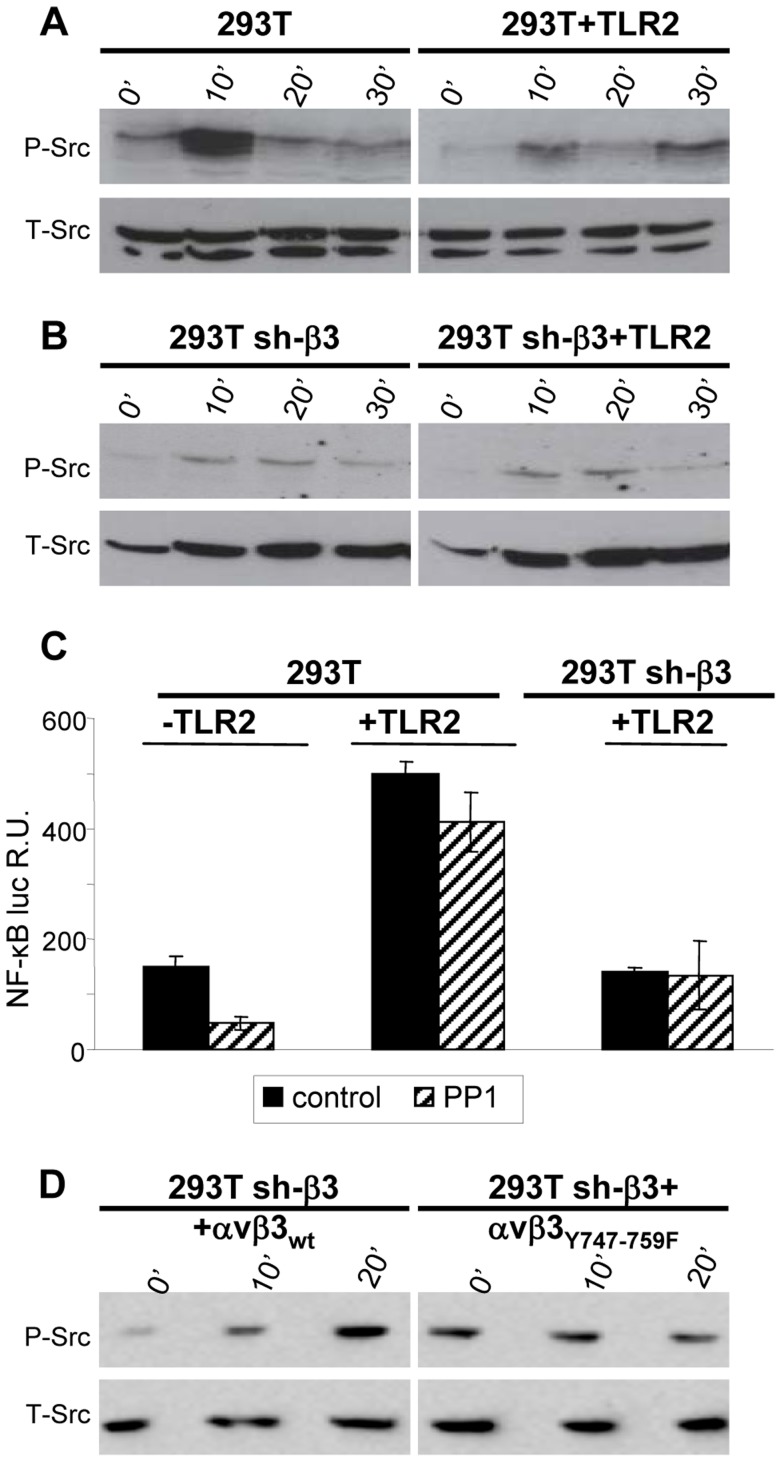
HSV-induced phosphorylation of Src, dependent on αvβ3-integrin. (A, B) 293T or 293T sh-β3 cells were transfected or not with plasmid encoding TLR2-Flag and exposed to R7910 (60 PFU/cell) for 10, 20 and 30 min. Cells were lysed; 300 ng of proteins were subjected to PAGE. The phosphorylated Src (P-Src) and total Src (T-Src) were detected with PAb to phospho-Src (Tyr416), or PAb 36D10 to total Src. (C) NF-κB activation is inhibited by PP1. 293T- or 293T sh-β3 cells were transfected or not with plasmids encoding TLR2, NF-κB-luc and Renilla luciferase. Cells were pre-treated with 1 µM PP1 for 1 h prior to exposure to gD^−/−^ virions and during virus absorption, or left untreated (control). Luciferase activity was quantified as detailed [8] in cells harvested at 6 h after infection. Each value represents the average of triplicate samples ± SD. (D) sh-β3 293T were transfected with wt-β3-integrin or β3_Y747-759F_, plus αv-integrin. Cells were cultured in pre-exhausted medium [8] for 48–72 h from 12 after transfection, and then exposed to R7910 (60 PFU/cell), or uninfected. Total cell lysates were subjected to SDS-PAGE and WB for phospho-Src (P-Src) or total SRC (T-Src) as loading control.

Having established that the αvβ3-integrin–TLR2 response entails an enhancement by αvβ3-integrin of the TLR2 signaling pathway, we asked the *viceversa* question, namely to what extent the αvβ3-integrin signaling branch contributes to the response. We focused on Src, because it belongs to a group of non-receptor tyrosine kinases typically activated by integrins. Furthermore, microarray analysis, validated by qRT-PCR, showed that some genes - *Src*, *Syk*, *Card9* - were activated *via* αvβ3-integrin, in a TLR2-independent fashion, by HSV infection or exposure to LPS [8]. Of note, this group of genes was upregulated to a modest extent (only 2–5 fold), whereas the IFN-α and -β genes were upregulated *via* the αvβ3-integrin–TLR2 axis about 100 fold. Here, we report that Src is phosphorylated very early - 10 minutes - following exposure of cells to HSV. The increase was much smaller in TLR2-positive cells than in TLR2-negative cells, hence it does not depend on TLR2 (Fig. 3 A). Further, the HSV-induced Src phosphorylation did not ensue in β3-integrin–silenced cells (Fig. 3 B), hence it depends on integrin alone, and not on the integrin–TLR2 cooperation. The functional role of Src was assessed through the effect of the PP1 (4-amino-5-(methylphenyl)-7-(t-butyl)pyrazolo-(3,4-d)pyrimidine) Src inhibitor on NF-κB activation induced by gD^−/−^ virions. When TLR2 was present a very modest inhibition (20%) was observed; in the absence of TLR2, the inhibition 70% (Fig. 3 C). Next, we investigated whether Src phosphorylation is dependent upon signaling by the C-tail of β3-integrin. sh-β3 cells expressing wt- β3 or β3-integrin_Y747-Y759_ were exposed to R7910 for 10 or 20 minutes, and endogenous phospho-Src was analyzed. An increase in phosphorylation was clearly detected in cells expressing the wt-β3-integrin, but not in cells expressing the mutant β3-integrin (Fig. 3 D), indicating that signaling by the β3-integrin C-tail targeted Src for phosphorylation. Together, the pattern of Src phosphorylation and the modest effect exerted by the Src inhibitor argue against an involvement of Src in the αvβ3-integrin–TLR2 concerted response, and, consequently, against an enhancement by TLR2 on the αvβ3-integrin signaling. The HSV-induced Src phosphorylation depends on αvβ3-integrin alone, in particular on signaling carried out by the C-tail.

### The NF-κB activation and IFN-β production dependent on αvβ3-integrin–TLR2 signaling involve Akt as a downstream hub

**Figure 4:**
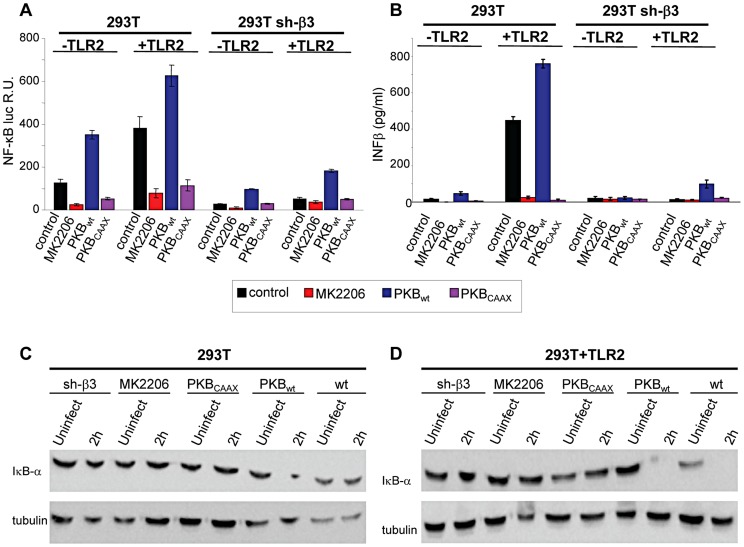
Inhibition od Akt reduces the innate response to HSV. (A–D) 293T- or 293T sh-β3 cells were transfected with plasmids encoding TLR2 or an empty plasmid, Akt-HA (PKB_wt_) or the dominant negative version PKB_CAAX_, as indicated. In lanes marked MK2206, cells were pre-treated with 5 µM MK2206 for 1 h prior to exposure to gD^−/−^ virions and during virus absorption, or left untreated (control), and harvested 6 h after infection. (A) Cells were additionally transfected with NF-κB-luc and Renilla luciferase. Luciferase activity was quantified as detailed in Fig. 1A [8]. Each value represents the average of triplicate samples ± SD. (B) Culture medium was harvested 48 h after infection. IFN-β was quantified as detailed in the legend to Fig. 1 D. (C, D), Total cell lysates were subjected to SDS-PAGE and blotted for IκB-α, or tubulin as loading control, as detailed in the legend to Fig. 1 C.

Akt constitutes a hub downstream of a number of signaling pathways. It participates in HSV-induced signaling cascades, both innate responses and downstream of FAK activation [21], [22]. Inasmuch as Src is not a station in the αvβ3-integrin–TLR2 concerted signalling, we asked whether Akt participates in it. We first performed functional assays, and asked whether the MK2206 Akt inhibitor reduced the HSV-induced NF-κB activation and IFN-β production. Cells were exposed to the inhibitor for 1 h prior to infection and during virus absorption. In preliminary experiments we employed R7910 and observed that NF-κB activation was practically abolished by 5 µM MK2206. Because, under these conditions, HSV infection was reduced to 50 and 40% in wt cells, and 5 and 30% in sh-β3 cells, and the reduction in NF-κB activation might reflect, in part, a reduction in virus entry, we made use of gD^−/−^ virions. Fig. 4 A and B show that the NF-κB activation and the IFN-β production induced by gD^−/−^ virions was almost abolished by MK2206. We also tested the effect of a DN version of Akt, named PKB-CAAX, which carries a CAAX motif derived from Ki-Ras [23]. Wt- or sh-β3 293T cells were transfected with the wt version PKB, plus ten-fold excess of PKB-CAAX, or with wt-PKB and 10-fold excess of empty vector, plus or minus TLR2. Cells were exposed to gD^−/−^ virions. Fig. 4 A and B show a dramatic reduction in NF-κB activation and IFN-β production by PKB-CAAX both in the absence and in the presence of TLR2. The reduction in activation of endogenous NF-κB by the above treatments was validated through measurements of IκB-α degradation, which paralleled the NF-κB activation seen in Fig. 4, panels A and B (Fig. 4 C, D).

### Akt phosphorylation is dependent on the αvβ3-integrin/TLR2 axis

**Figure 5:**
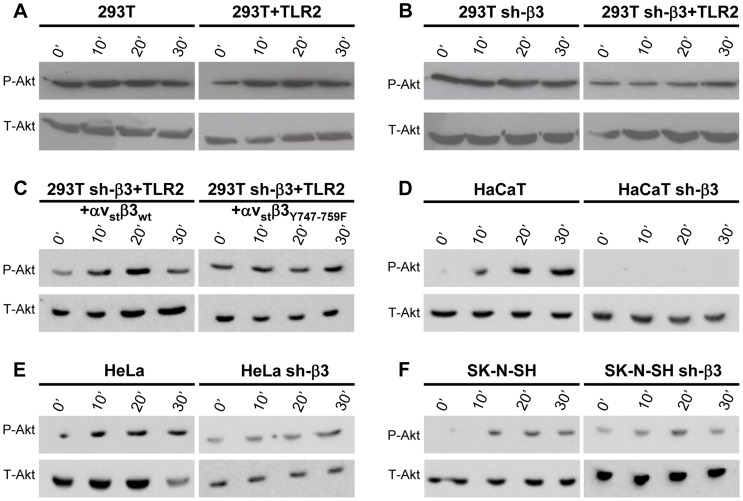
HSV-induced Akt phosphorylation dependent on αvβ3-integrin, its C-tail, and TLR2. (A, B, D–F) Akt phosphorylation requires β3-integrin and TLR2. wt or sh-β3 cells 293T (A, B), HaCaT (D), HeLa (E), SK-N-SH (F) cells were transfected or not with plasmid encoding TLR2-Flag, silenced for β3-integrin as indicated (sh-β3), exposed to R7910 (60 PFU/cell) for 10, 20 and 30 min. Cells were lysed and total proteins were subjected to SDS-PAGE. Phosphorylated and total Akt were detected with PAb to phospho-Akt (Ser473) (P-Akt) or PAb to total Akt (T-Akt). (C) β3-integrin silenced (sh-β3) 293T were transfected with wt-β3-integrin or β3_Y747-759F_ plus αv-integrin, and TLR2. Cells were cultured in pre-exhausted medium [8] for 48–72 h from 12 after transfection, and then exposed to R7910 (60 PFU/cell) for 10, 20, 30 min, or uninfected (0′). Total cell lysates were subjected to SDS-PAGE and WB for P-Akt or T-Akt.

Next, we verified whether Akt undergoes phosphorylation, and whether the phosphorylation occurs in αvβ3-integrin/TLR2-dependent fashion. Fig. 5 A and B show the extent of phospho-Akt (Ser473) (P-Akt) and total Akt (T-Akt), as determined by western blotting (WB), following exposure of wt-293T or sh-β3 cells to R7910 for 10, 20 or 30 min. In these cells, Akt was overall modestly phosphorylated. Yet, the phosphorylation was consistently observed in numerous experiments. The HSV-induced increase occurred in the presence of TLR2 (Fig. 5 A), was overall lower in sh-β3 cells (Fig. 5 B). We next verified whether Akt phosphorylation was dependent on the signaling activity carried out by the C-tail of β3-integrin. The sh-β3 cells, transfected with wt-β3- integrin or C-tail integrin mutant were exposed R7910 for 10, 20, 30 min. Fig. 5 C shows that Akt phosphorylation was increased more than two-fold at 20 minute in cells expressing wt-β3-integrin; such increase was not seen in cells expressing the C-tail β3-integrin mutant. The pattern of Akt phosphorylation in 293T cells indicates that it is TLR2-dependent, and, in part, β3-integrin–dependent. This pattern of Akt phosphorylation - dependent on the αvβ3-integrin–TLR2 axis - was markedly different from that of Src phosphorylation.

We checked the Akt involvement in the αvβ3-integrin/TLR2 signaling axis in cells other than 293T by analysis of Akt phosphorylation. The selected cells were those analysed in Fig. 2 for the αvβ3-integrin–enhanced MYD88 recruitment to TLR2. HaCaT, HeLa, and SK-N-SH cells were silenced for β3-integrin, or non-silenced, and exposed to R7910 for 10, 20, 30 min. In all wt-cells, Akt underwent phosphorylation, or an increase in phosphorylation, upon exposure to R7910 (Fig. 5 D–F). The increase was seen irrespectively of the differences in the basal level of Akt phosphorylation seen in unexposed cells. The virus-induced increase in Akt phosphorylation was not seen in the β3-integrin-silenced cells (Fig. 5 D–F, right panels). Thus, in cells of different origin, the αvβ3-integrin/TLR2 signaling axis involves Akt as a downstream hub.

### R7910 replication is rescued in cells where the αvβ3-integrin/TLR2 signaling cascade is hampered by expression of the DN MYD88_FW/AA_ or PKB_CAAX_

**Figure 6:**
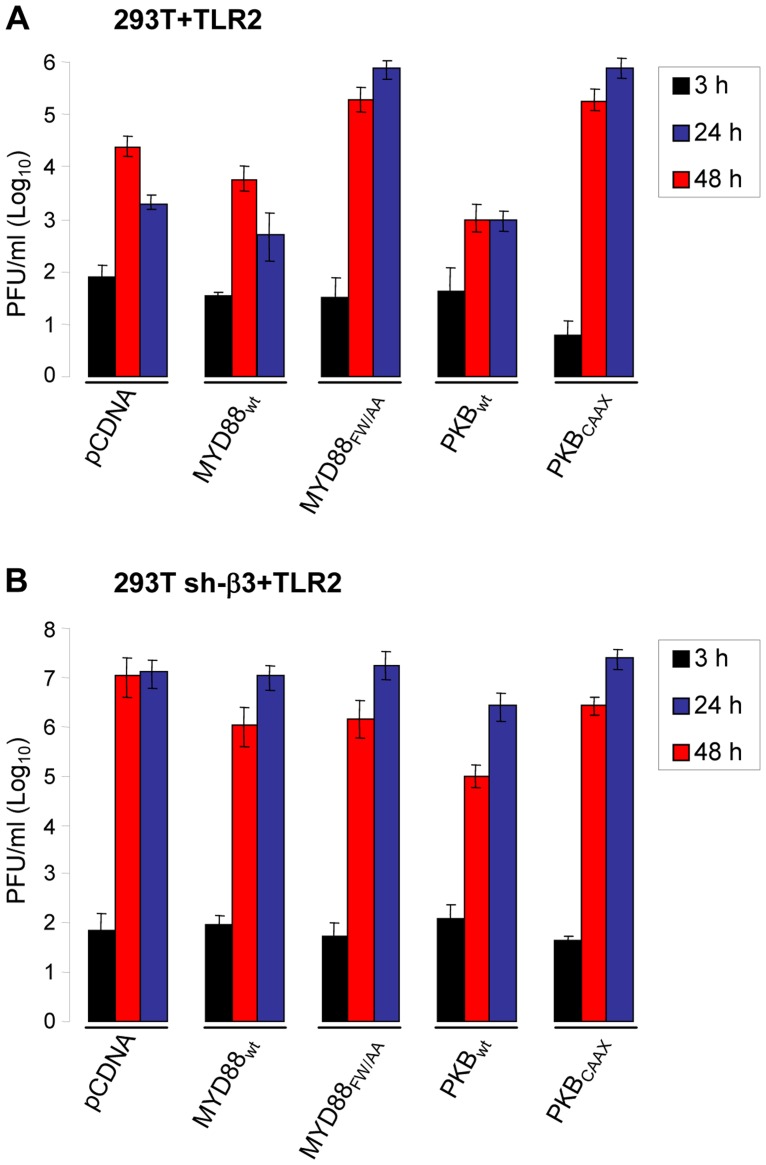
R7910 yield in cells expressing DN versions of MYD88 or AkT, and silenced for β3-integrin. 293T (A) or 293T sh-β3 (B) cells were transfected with the indicated plasmids, or pCDNA, as control, plus TLR2. Cells were infected with R7910 (1 PFU/cell), and harvested 3, 24, 48 h after infection for titration of progeny virus in U20S cells. Each value represents the average of triplicate samples ± SD.

HSV mutants deleted or mutated in ICP0 are strongly defective in replication, because they are defective in counteracting a number of the host defences. To shed light on the significance of the αvβ3-integrin/TLR2 response to the virus cycle, we measured R7910 replication in cells where the pathway was impaired through expression of DN mutants MYD88_FW/AA_ or PKB_CAAX_, through silencing of β3-integrin, or both. Wt 293T or 293T sh-β3 cells were transfected with the wt or DN forms of the intermediates. Cells were infected with R7910, and progeny virus titrated at 24 or 48 h after infection. Fig. 6 A shows that MYD88_FW/AA_ or PKB_CAAX_ rescued R7910 yield by about 2 Lg or more. The rescue was even higher in cells silenced for β3-integrin (Fig. 6 B), in accordance with previous data [8]. Clearly, the αvβ3-integrin/TLR2 response is highly detrimental to the replication of the ICP0-minus HSV.

### gH is the PAMP of the αvβ3-integrin–TLR2 system

**Figure 7:**
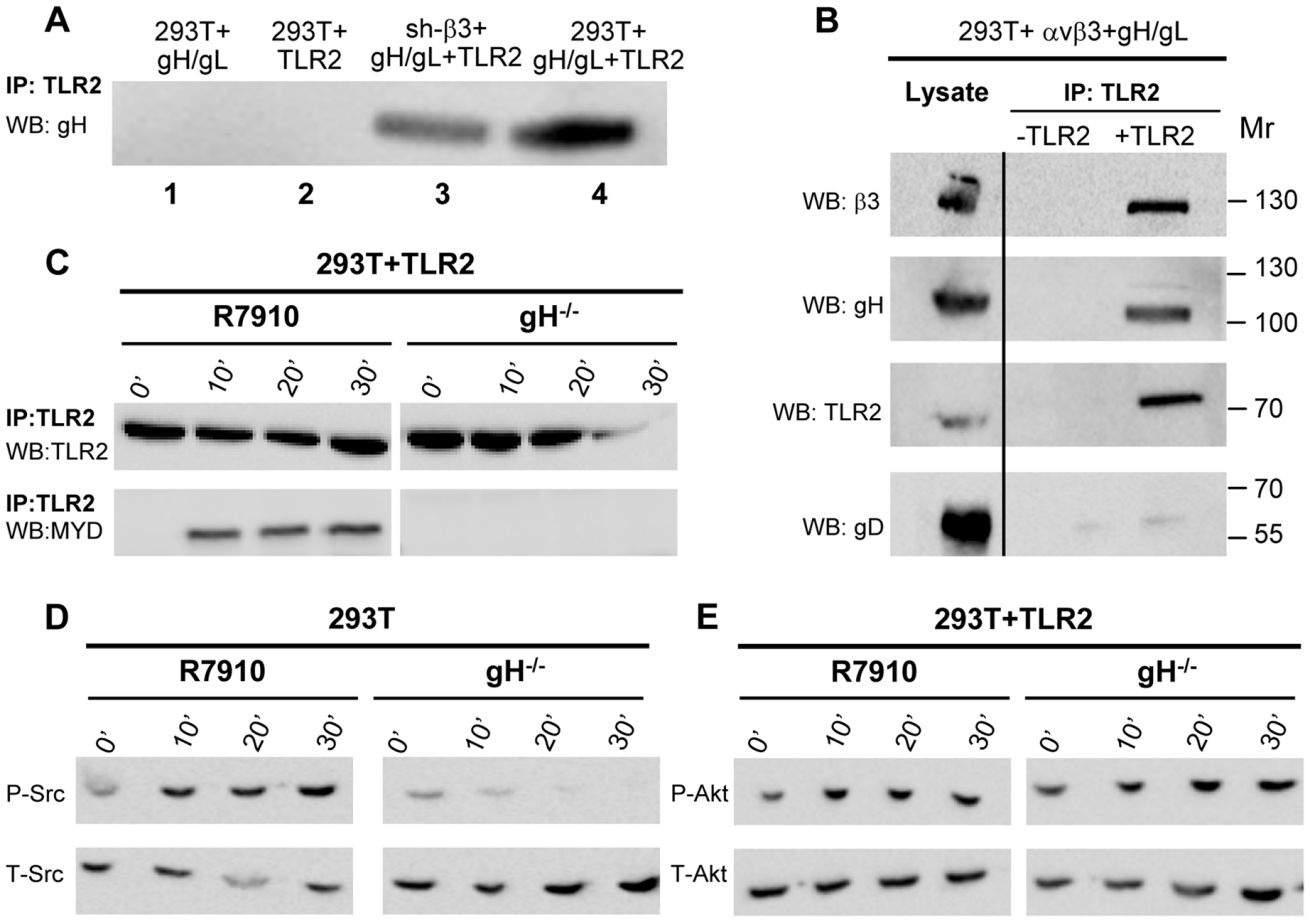
Interaction of gH with TLR2 and β3-integrin, and failure of gH^−/−^ virions to elicit the innate response. (A) Co-immunoprecipitation of gH by TLR2 occurs in β3-integrin–silenced cells. 293T cells were transfected with plasmids encoding gH/gL (lane 1), TLR2-Flag (lane 2), gH/gL plus TLR2-Flag (lane 4). sh-β3 293T cells were transfected with plasmids encoding gH/gL plus TLR2-Flag (lane 3). TLR2-Flag was immunoprecipitated with anti-Flag MAb (IP: TLR2). The co-immunoprecipitated gH was revealed by WB with PAb to gH/gL. (B) 293T cells were transfected with plasmids encoding TLR2-Flag, αv-integrin+β3-integrin, gH+gL. Negative controls lacked TLR2. TLR2-Flag was immunoprecipitated with anti-Flag MAb (IP: TLR2). The co-immunoprecipitated gH and β3-integrin were revealed by WB with PAb to gH/gL, MAb to TLR2-Flag, and PAb to β3-integrin. Figures to the right indicate the migration position and M_r_ of molecular weight markers. (C–E) Failure of gH^−/−^ virions to induce the MYD88 recruitment to TLR2 (C), phosphorylation of Src (D), and phosphorylation of Akt (E). 293T cells, transfected or not with TLR2-Flag, were exposed to R7910 or gH^−/−^ virions for 10, 20, 30 min, or unexposed (0′). (C) TLR2-Flag was immunoprecipitaed with anti-Flag MAb. Co-immunoprecipitated MYD88 was detected by WB, as detailed in the legend to fig. 2A. (D, E) Total cell lysates were subjected to SDS-PAGE and WB for P-Src and T-Src (D), or for P-Akt and T-Akt (E).

A soluble form of gH/gL interacts physically with a soluble form of αvβ3-integrin at 10^−6^ M affinity [8]. We reported that in αvβ3-integrin–positive cells, TLR2 can co-immunoprecipitate gH/gL [9]. Here, we asked whether the observed gH/gL– TLR2 interaction was mediated by αvβ3-integrin, or was independent of it. We expressed gH/gL in αvβ3-integrin–positive or in sh-β3 cells, in the absence or presence of TLR2-Flag. Fig. 7 A shows that gH/gL was co-immunoprecipitated by TLR2 in β3-integrin-silenced cells (lane 3), as well as in wt 293T cells (lane 4). Hence, gH/gL interacts with TLR2 independently of αvβ3-integrin. We verified that gH/gL indeed cross-links αvβ3-integrin and TLR2-Flag. 293T cells were simultaneously transfected with TLR2-Flag, αv+β3-integrin, gH, gL. The controls were devoid of TLR2. TLR2-Flag was immunoprecipitated. Fig. 7 B shows that TLR2-Flag coimmunoprecipitated both gH and β3-integrin, but not gD. Altogether, αvβ3-integrin and TLR2 interact one with the other under resting conditions, i.e. in the absence of ligands, [6], [8]. In addition, gH/gL can interact with TLR2 and with αvβ3-integrin, independently one of the other, and indeed gH/gL recruit both of them to a complex, and cross-link them.

To provide further evidence in support of gH/gL as the PAMP of the αvβ3-integrin/TLR2—mediated innate response, we checked whether gH^−/−^ virions are defective in MYD88 recruitment to TLR2, and in Src and Akt phosphorylation. gH^−/−^ virions are deleted in gH [24] and were grown in non complementing cells. They can attach to cells but fail to infect them. The response elicited by these virions represents the immediate innate response to incoming virions, prior to their fusion with the target cells. Cells were exposed to gH^−/−^ virions, and, for comparison, to R7910. In cells exposed to gH^−/−^ virions, TLR2 failed to recruit MYD88 (Fig. 7 C); Src phosphorylation was almost completely abolished (Fig. 7 D); the increase in Akt phosphorylation was moderately reduced in TLR2^+^293T as compared to that elicited by R7910, in agreement with the modest Akt activation seen in these cells (Fig. 7 E). Together, the previous finding that gH^−/−^ virions fail to elicit NF-κB response [9], the ability gH/gL and of αvβ3-integrin to interact with TLR2, the defect of gH^−/−^ virions to induce the MYD88 recruitment to TLR2, and Akt phosphorylation argue for gH/gL as the HSV PAMP of the αvβ3-integrin/TLR2 system.

### CD14 augments the αvβ3-integrin–TLR2 mediated NF-κB response but does not substitute for αvβ3-integrin in enhancing the TLR2 response

**Figure 8:**
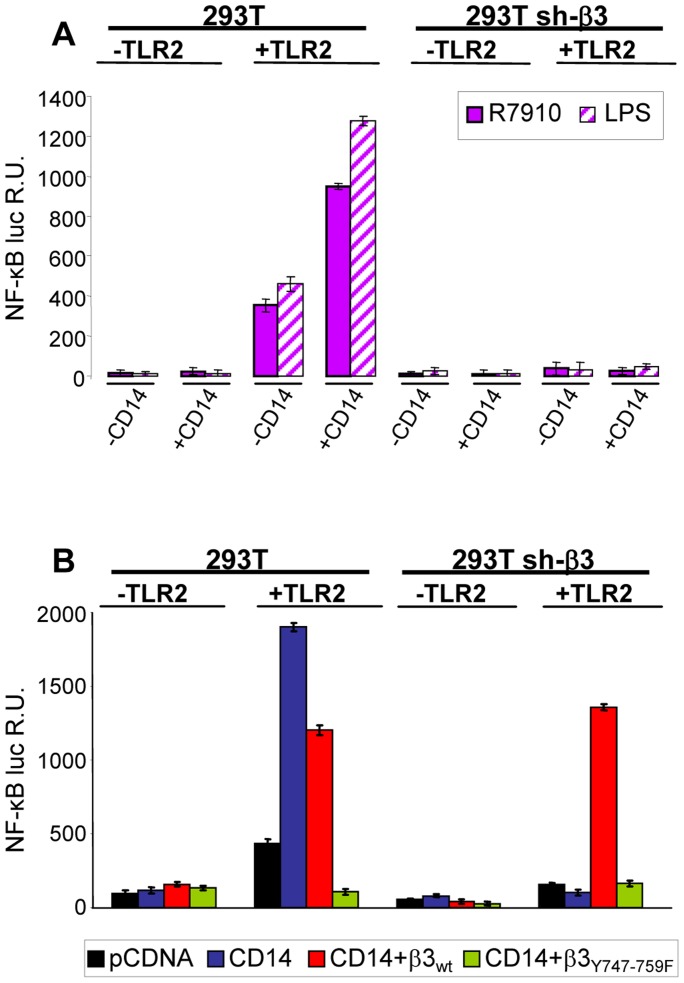
CD14 augments the NF-κB response triggered by αvβ3-integrin and TLR2. (A) 293T- or sh-β3 293T cells were transfected with plasmids encoding TLR2, or empty plasmid, NF-κB-luc and Renilla luciferase, ± CD14, as indicated. Cells were then infected with R7910 (20 PFU/cell) or exposed to LPS (100 ng/ml) for 4 h. (B) 293T or 293T sh-β3 cells were transfected with plasmids encoding TLR2, CD14, wt-β3-integrin or β3_Y747-759F_, as indicated, plus NF-κB-luc and Renilla luciferase, All other details as in panel A. In A and B, NF-κB-luc was quantified as detailed [8]. Each value represents the average of triplicate samples ± SD.

CD14 is a costimulatory protein or co-receptor for a number of TLRs, including TLR2 [5], [19], [25]. It also enhances the HSV-induced TLR2 response [4]. Having established that the αvβ3-integrin–TLR2 concerted activity occurs through an enhancement by integrin of the TLR2 cascade, we asked whether CD14 plays a role in it. In particular, we asked whether CD14 augments the αvβ3-integrin–TLR2 concerted response, or whether CD14 can substitute for αvβ3-integrin in enhancing the TLR2 response. wt or β3-integrin-silenced 293T cells were transfected with CD14, TLR2-Flag, or both, plus NF-κB-luc and Renilla luciferase, and induced with R7910 for 6 h, or LPS for 4 h. Fig. 8 A shows that CD14 increased the integrin–TLR2 mediated response by about 2.5 fold. CD14 had no effect on the silenced cells (right panel), not even on those expressing TLR2, indicating that it does not substitute for αvβ3-integrin in enhancing the TLR2 response. Further, we investigated whether the CD14-mediated enhancement of the NF-κB response involved the C-tail signaling portion of β3-integrin. In sh-β3 cells expressing the β3-integrin_Y747-Y759_ mutant the CD14-mediated enhancement of NF-κB response was almost abolished (Fig. 8 B). Altogether, the enhancement by CD14 of the αvβ3-integrin–TLR2 response strengthens the above conclusion that integrin–TLR2 cooperation rests on boosting of the TLR2 signaling by integrin, in particular by its C-tail.

### Lipid rafts serve as platforms for the αvβ3-integrin-dependent re-localization of TLR2, MAL and MYD88

**Figure 9:**
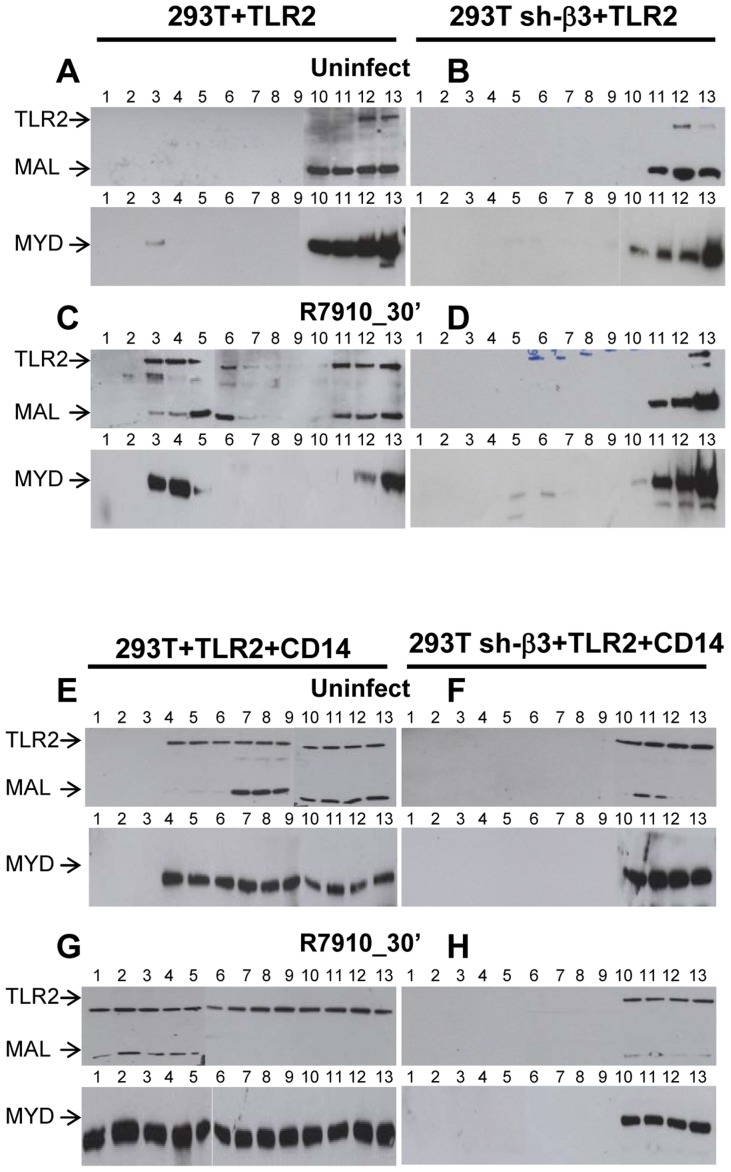
Lipid rafts serve as platforms for the αvβ3-integrin-dependent re-localization of TLR2, MAL and MYD88. 293T or 293T sh-β3 cells were transfected with plasmids encoding TLR2-Flag, MYD88-HA and MAL-Flag plasmids. When indicated, CD14 plasmid was included (E–H). 48 h after transfection, cells were exposed for 30 min to R7910 (60 PFU/cell) (C, D, G, H) or mock-infected (A, B, E, F). Cells were suspended in 1 ml of TNE buffer (10 mM Tris-HCl, pH 7.5, 150 mM NaCl, 5 mM EDTA) containing 1% Triton X-100 (Sigma Aldrich, Milan, Italy) and 0.3 mM protease inhibitors, and incubated on ice for 1 h. Membrane fractions were prepared as described [26]. The samples obtained from fractionation of the sucrose gradients (# 1–13) were subjected to SDS-PAGE and WB with MAb to HA for detection of MYD88, and MAb to Flag for detection of TLR2 and MAL.

We asked which is the subcellular compartment where the αvβ3-integrin–enhanced recruitment of MYD88 to TLR2 takes place, and, in particular, whether the TLR2-MYD88 complex is assembled at or around lipid rafts. Wt or sh-β3 293T cells were transfected with TLR2-Flag and MYD88-HA. MAL, the connector between TLR2 and MYD88, was included. Cells were exposed to R7910 for 30 min. Membranes were fractionated, and allowed to float in sucrose gradients. Previously, we showed that the top light fractions of the gradient contain molecules typical of lipid rafts, e.g. GPI-anchored receptors [26]. The middle fractions contain molecules that localize at or around lipid rafts. The bottom fractions contain the heavy membrane fractions. Fig. 9 shows the immunoblot analysis of gradient-partitioned membranes. Prior to cell exposure to virus, TLR2, MYD88 and MAL partition with the heavy fractions of the gradient (Fig. 9 A). Following exposure to R7910, a portion of TLR2, MYD88 and MAL partitions at light-middle fractions of the gradient (Fig. 9 C), indicating that HSV induces a relocalization of these molecules at or around lipid rafts. The re-localization required integrin, since it did not occur in sh-β3 cells (Fig. 9, B and D). The results indicate that αvβ3-integrin participates in the initiation of the TLR2 signaling also by promoting the relocalization of TLR2, MAL, MYD88. Likely, the relocalization favours complex assembly.

We next examined the contribution of CD14 to the lipid raft localization of the above molecules. The above experiment was repeated in the presence of CD14. Fig. 9 E shows that, in the presence of CD14, a portion of TLR2, MAL, MYD88 partition to the middle fractions, i.e. are localized at or around lipid rafts, prior to exposure of cells to HSV. Exposure of cells to HSV further increases the lipid raft localization (compare panel G to panel E). The HSV-induced compartimentalization to lipid rafts does not occur in sh-β3 cells (Fig. 9, F, H), indicating that it requires αvβ3-integrin.

#### Functional perturbation of lipid rafts abolishes the HSV-induced NF-κB and IFN-β response

**Figure 10:**
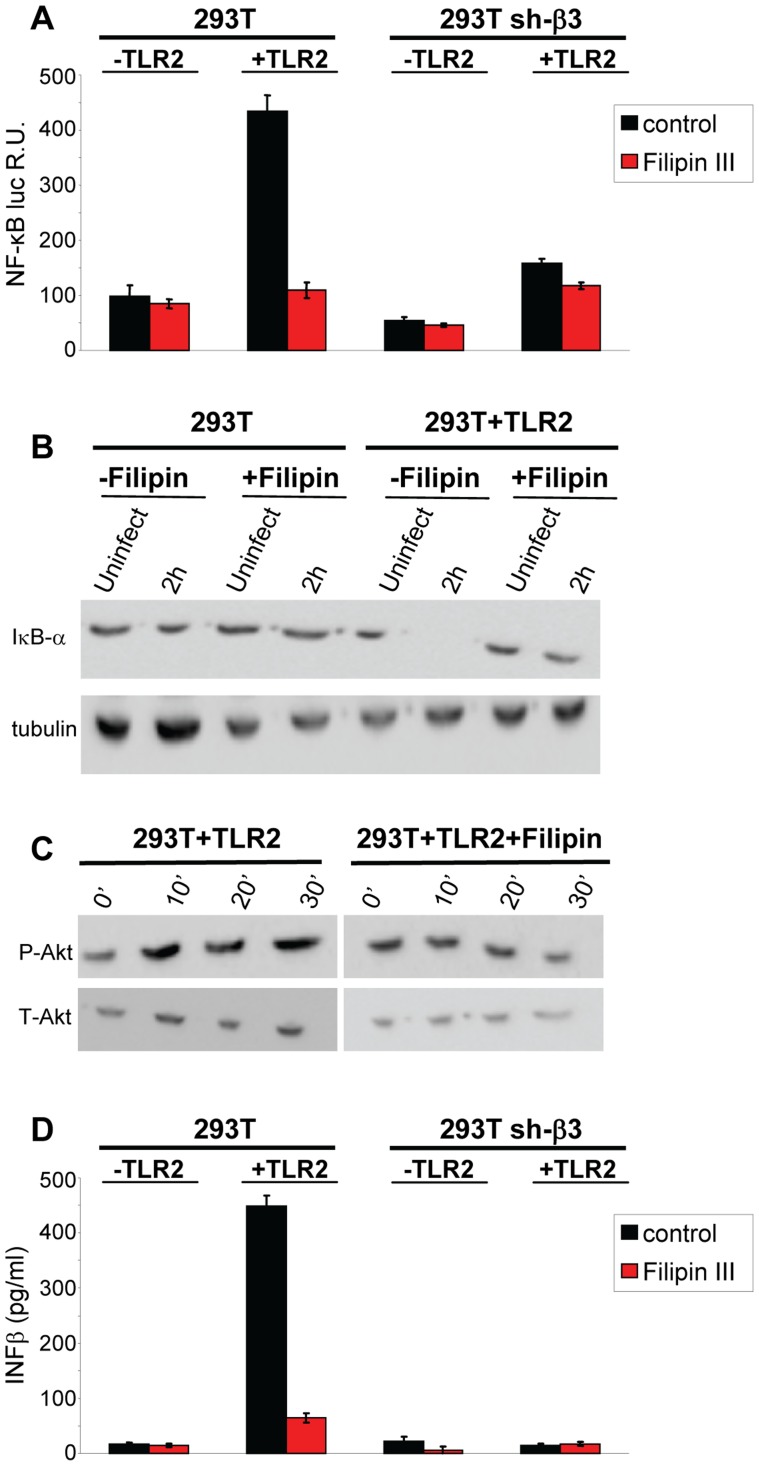
Lipid rafts perturbation by the cholesterol-binding filipin III blocks the innate response to HSV. (A, B) Effect of filipin III on NF-κB activation. (A) 293T- or sh-β3 293T cells were transfected with plasmids encoding TLR2, NF-κB-luc and Renilla luciferase, as indicated. Total cell lysates were subjected to SDS-PAGE and WB for IκB-α, or tubulin (panel B), as detailed in the legend to Fig. 1 C, or for phospho-Akt and total Akt (panel C), as detailed in the legend to Fig. 5. (D) Wt 293T (293T) or sh-β3 293T cells were transfected with TLR2 as indicated. IFN-β was quantified in the culture medium 48 h after infection, as detailed in the legend to Fig. 1 D. In A and D, each value represents the average of triplicate samples ± SD. In all panels filipin III treatment consisted of exposure of cells to the compound (1.5 µM) from 30 min prior to virus absorption till the end of virus absorption with gD^−/−^ virions, at 20 PFU equivalent/cell (panels A, B, D) or 60 PFU equivalent/cell (panel C). Cells were harvested at the indicated times, 6 h in panel A, 2 h in panel B; 10, 20, 30 min in panel C; 48 h in panel D. All other details as in the legend to Fig. 1A.

To provide a functional assay for the involvement of lipid rafts in the HSV-induced NF-κB and IFN-β response, we made use of filipin III, a cholesterol-sequestering compound which impairs lipid raft function as organizing centres of signaling molecules. Because filipin III inhibits HSV infection [27], we made use of gD^−/−^ virions. We tested the effect of filipin III treatment on the NF-κB-luc response, on degradation of endogenous IκB-α, on Akt phosphorylation, and on IFN-β production elicited by gD^−/−^ in TLR2^+^ 293T cells. Fig. 10 A–D shows that exposure of cells to filipin III, 30 min prior to infection and during virus absorption, practically abolished the NF-κB-luc activation induced by gD^−/−^ virions (Fig. 10 A), the degradation of endogenous IκB-α seen at 2 h after cell exposure to virus (Fig. 10 B), the Akt phosphorylation (Fig. 10 C), and IFN-β secretion at 48 h. The results consistently show that the compartmentalization of the molecules to lipid rafts, dependent on αvβ3-integrin, is a critical event in activation of the αvβ3-integrin/TLR2 signaling cascade and response.

## Discussion

**Figure 11:**
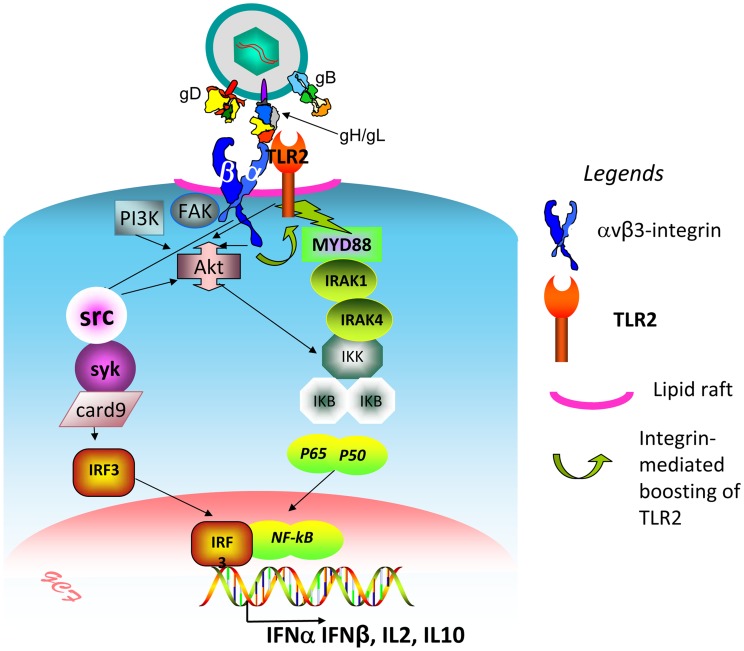
Simplified view of the concerted contribution of αvβ3-integrin and TLR2 to the immediate defensive response of the cell to an invader, exemplified by HSV. The virion envelope gH bind simultaneously αvβ3-integrin and TLR2, and thus cross-links them. Signaling by the C-tail of the β3 subunit (green curved arrow) results in boosting of the TLR2-dependent pathway – MAL, MYD88 recruitment, IRAK1 and 4 phosphorylation, and NK-κB activation. Akt serves as a hub, downstream of αvβ3-integrin and TLR2, possibly through the prior activation of FAK and PI3K. Src, and, downstream, syk, card9, IRF3 are activated by αvβ3-integrin, independently of TLR2. The major cellular targets of this pathway are IFN-α, IFN-β, and a specific set of cytokines, including IL2 and IL10.

We investigated how αvβ3-integrin and TLR2 act in concert to elicit the immediate branch of the innate response to HSV and to LPS. The key findings to emerge are that (i) αvβ3-integrin boosts the MYD88-dependent TLR2 signaling and defensive response; this was seen in all cell lines tested, i.e. the model 293T, and the keratinocytic, epithelial and neuronal cell lines, which are models of the HSV targets *in vivo*. In contrast, TLR2 exerts no effect on the αvβ3-integrin signaling pathway. (ii) The herpes simplex virion glycoproteins gH/gL serve as the PAMP and cross-link the two triggering receptors, (iii) αvβ3-integrin and TLR2 are relocated to lipid rafts in a ligand (virion)-dependent fashion; (iv) Akt serves as a hub of the signaling pathway (see, Fig. 11 for a schematic overview).

The initial investigations provided evidence that the basis of the concerted αvβ3-integrin–TLR2 action rests on signaling activity, and not on enhancement of HSV entry, or LPS binding/uptake, since the NF-κB activation, IFN-β secretion, and recruitment of MYD88 to TLR2 were strongly impaired when wt β3-integrin was replaced with a mutant carrying substitutions in the cytoplasmic tail, which prevent phosphorylation and signaling. Moreover, the gD^−/−^ virions, competent for attachment but not for entry, triggered a response which required the signaling portion of β3-integrin C-tail, hence similar to that elicited by the replication competent HSV. This indicated that entry of HSV into the cell is not a requirement to initiate the immediate branch of the response mediated in concert by αvβ3-integrin and TLR2. This response clearly differs from the one elicited by fusion of HS virions with the cell [28]. Numerous studies highlighted that the cell response to HSV can be differentiated in at least two temporal waves. The first, immediate response is that exerted by UV-inactivated (able to enter cells but unable to express viral genes) HSV, exemplified in current studies by gD^−/−^ virions. They activate NF-κB and other lines of defence within a few minutes after infection [29], [30], along with activation of FAK [31]. More sustained NF-κB activation and cell defence occur at later times under the stimulus of additional viral gene products, including U_S_3, VP11/12, U_L_31 [32]–[37]. While all these gene products play critical roles in eliciting the global cell defence, nonetheless, previous and current studies from our laboratory indicate that the response elicited by the αvβ3-integrin/TLR2 axis make a significant contribution also to the second wave of defence. Indeed, the absence of TLR2, the silencing of β3-integrin, or both, dramatically decreased the NF-κB activation, and, importantly, the IFN-α and -β production [8].

We dissected the pathways downstream of αvβ3-integrin and downstream of TLR2. The key findings were that αvβ3-integrin boosts the TLR2 signaling, which typically involves recruitment of TIRAP/MAL, MYD88, followed by phosphorylation of IRAK1 and IRAK 4 [19]. In β3-integrin-silenced cells (293T, HaCaT, HeLa and SK-N-SK), or in 293T cells expressing the C-tail mutant of β3-integrin, MYD88 was not recruited to TLR2, and consistently, IRAK 4 phosphorylation was almost abolished in β3-integrin-silenced 293T cells. Additional support for a functional role of the MYD88 was provided by a DN version of this molecule (MYD88_FW/AA_), capable to be recruited to TLR2 but unable to signal downstream [20]. MYD88_FW/AA_ drastically reduced NF-κB activation. HSV counteracts the αvβ3-integrin/TLR2 antiviral response, soon after it enters the cell, at the onset of viral protein synthesis by means of the immediate early protein ICP0 [8]. ICP0 alone can reduce the levels of MYD88 and MAL [38], a property that lends indirect support to the αvβ3-integrin–mediated boosting of TLR2 signaling.

The converse effect, i.e. the enhancement by TLR2 of the αvβ3-integrin signaling response was not detected. In particular, we ruled out an effect of TLR2 on the activation of the non receptor tyrosine kinase Src. This was the most appropriate candidate to be examined downstream of αvβ3-integrin, since Src is one of the most typical molecules activated in the integrin signaling pathway [39], and, mainly, because *Src*, together with *Syk* and *Card9*, were upregulated by HSV in an αvβ3-integrin-dependent TLR2-independent fashion in microarray analysis [8].

To further elucidate the signaling pathways downstream of the αvβ3-integrin–TLR2 axis, we considered Akt, a molecule on which several signaling pathways converge, and known to be involved in HSV infection [21], [22]. Akt serves as a hub in the αvβ3-integrin–TLR2-mediated signaling. Thus, upon HSV infection, or LPS stimulation, Akt was phosphorylated in β3-integrin-integrin-silenced 293T, HaCaT, HeLa, SK-N-SK cells, in a TLR2-dependent fashion, in cells expressing the DN version of AKT (PKB-CAAX) [23], or in cells exposed to the MK2206 specific inhibitor.

gH/gL are essential glycoproteins in the process of HSV entry into the cells [15]. They are part of the conserved fusion apparatus across the *Herpesviridae* family, and transmit the activation signal which ultimately activates the fusion glycoprotein gB [14]. We identified the virion envelope glycoprotein gH/gL as the HSV PAMP of this system, based on three lines of evidence. First, interactions were documented here and elsewhere between gH/gL and TLR2, or between gH/gL and αvβ3-integrin, independently one (see Fig. 5 and references [6], [8], [9]. The latter was also documented between the virion gH/gL with αvβ3-integrin [40]. In addition, αvβ3-integrin and TLR2 interact one with the other under resting conditions, in the absence of ligands [6], [8]. Secondly, we observed that gH/gL cross links the two receptors. Furthermore, gH^−/−^ virions were defective in key steps of the αvβ3-integrin/TLR2 signaling, including the recruitment of MYD88 to TLR2, and Akt phosphorylation. We propose that the gH/gL-mediated cross-linking of αvβ3-integrin and TLR2 represents the starting event in the activation of the signaling cascade. This mechanism differs from the αvβ3-integrin–TLR2 cooperation described by Gerold *et al* in response to lipopeptide [6]; in that case, the role of αvβ3-integrin was to bind the lipopetide and to present it to TLR2, whereas in our system gH/gL cross-links the two receptors. It is important to note that, even though we have focused our studies mainly on the interaction of HSV with αvβ3-integrin and TLR2, the main results were obtained also with LPS. Thus, HSV gH/gL should not be considered as the only PAMP in this system, and, in turn, this defensive system should not be considered as exclusively devoted to anti-viral activity.

CD14 is a GPI-anchored accessory molecule, or coreceptor for a number of TLRs, including TLR2. Its serves several functions, including to facilitate the presentation of LPS to TLR2, TLR2 heterodimerization, the ligand-induced localization of TLR2 to lipid rafts, and the MYD88-independent signaling of TLR4 [5], [19], [25]. We found that CD14 can boosts the TLR2 signaling in an additive manner relative to αvβ3-integrin, but can not substitute for integrin, since it failed to exert any enhancing effect in cells in which β3-integrin was silenced, or mutated. Hence, a clear hierarchy between CD14 and αvβ3-integrin exists: αvβ3-integrin boosts TLR2 signaling irrespective of the absence or presence of CD14. CD14 boosts the TLR2 response only in the presence of integrin. The boosting effect of CD14 on TLR2 response described here differs from that seen in macrophages, a system in which CD14 was a requirement [41].

From a mechanistic point of view, αvβ3-integrin relocates TLR2, MAL and MYD88 at or around lipid rafts. CD14 further enhances both the ligand–independent and –dependent lipid raft localization of the receptors. The functional perturbation of lipid rafts abolished the HSV-induced NF-κB activation and IFN-β response. Of note, αvβ3-integrin relocates also relocates the HSV receptor nectin1 to lipid rafts [42]. Altogether, lipid rafts represent the platforms at, or around which the αvβ3-integrin-enhanced recruitment of MYD88 to TLR2 takes place. Under this respect, αvβ3-integrin behaves similarly to CD14 and CD36 coreceptors [43], [44].

A key result is that this branch of the innate response is highly detrimental to the virus. R7910 replication was dramatically increased (2–4 Logs) in cells in which MYD88 or Akt were replaced by DN mutants, and, especially in cells where β3-integrin was silenced and MYD88 or Akt were simultaneously replaced with DN mutants. We conclude that the cell deploys the αvβ3-integrin/TLR2 mediated response as a defensive antiviral system.

The key finding of this work, that αvβ3-integrin boosts the MYD88-dependent TLR2 signaling (see schematic overview in Fig. 11), is best interpreted in the context of coreceptors that reinforce or broaden the activity of TLR2, and their cell-type distribution. Among the cell surface TLRs, TLR2 emerges as the one capable to recognize a wide range of molecular patterns. The broad repertoire of exogenous and endogenous molecular patterns may well be achieved through interaction with co-receptors. In addition to CD14 mentioned above, which is preferentially expressed in monocytic cells, TLR2 coreceptors include CD36, a member of the scavenger receptor family preferentially expressed in monocytic and endothelial cells [45]–[47], certain leukocyte- monocyte-specific integrins, e.g. αMβ2-integrin and α3β1-integrin in monocytes-macrophages [5], [19], [48], [49]; αMβ2-integrin positively regulates TLR4 in in dendritic cells [50]. Previously, we reported that the concerted αvβ3-integrin–TLR2 response represents a major innate response elicited by HSV in epithelial, keratinocytic and neuronal cell lines, i.e. in cells which are models of the cells targeted by HSV *in vivo* [7]. The silencing of β3-integrin in these same cells results in inhibition TLR2 signaling, seen as inhibition of MYD88 recruitment to TLR2. Given the wide distribution of αvβ3-integrin in epithelial cells, we propose that αvβ3-integrin may well serve as the coreceptor employed by TLR2 in these cells, and what so far was described as the TLR2 response in epithelial cells is very likely the concerted αvβ3-integrin–TLR2 response. In essence, the role of the widely expressed αvβ3-integrin was so far simply unnoticed. Taken together, current and previous data argue that in several cell systems TLR2 actually requires one or another coreceptor, and that the coreceptors likely contribute to the cell type specificity and broad spectrum of the TLR2 response.

## Materials and Methods

### Cells and viruses

293T, HeLa, SK-H-SH and U20S cells were received from American Type Culture Collection and grown in Dulbecco's modified Eagle's medium containing 10% foetal bovine serum. HaCaT cells were received from Deutsches Krebsforshungzentrum, Heidelberg, and grown in high (4%) glucose RPMI containing 10% foetal bovine serum. β3-integrin silenced 293T cells (named sh-β3) and the β3-integrin silenced HaCaT, HeLa, SK-H-SH (named HaCaT sh-β3, HeLa sh-β3, SK-H-SH sh-β3) were described [7], [8]. R7910 is a HSV mutant deleted in the gene encoding ICP0 [18]. The wRR-1097 gD deletion HSV was described [51]. The gH deletion HSV was described [24].

### Plasmids

The mammalian expression plasmids encoding HSV-1 gH, gL, gD,under the cytomegalovirus promoter, were described [52]. The αv, β3_wt_, β3_Y747F_, β3_Y759F_ and β3_Y747-759F_ expression plasmids were a generous gift from Dr. Blystone [17]. Plasmids encoding TLR2-Flag and NF-κB-luc were a generous gift from Dr. D.M. Knipe [4]. pCMV-HA-MYD88, pCMV-HA- MYD88_FW/AA_, pEFBos-MAL-Flag and CD14 in pCDNA were form Addgene. pcDNA3.1 was from Invitrogen. Renilla luciferase plasmid was from Promega. Plasmids encoding HA-PBK and PKB-CAAX were generous gifts from Dr. B. Burgering [23]. The plasmid encoding strep-tagged αv-integrin (named αv_st_ or αv_strep_) was generated as follows. αv-integrin was excised from pcDM8 by insertion of two restriction sites, one at the 5′ (NotI) and one at 3′ end (BamHI), just before the stop codon. The mutagenic oligonucleotides were 5′-gcttggcgtcccgcgGCcGcttcggcgatggcttttcc-3′ and 5′-ggaaaagccatcgccgaagCgGCcgcgggacgccaagc-3′, and 5′- ggtgaaggaaactcagGGaTCCaactgcagtttttaagttatgc-3′ and 5′- gcataacttaaaaactgcagttGGAtCCctgagtttccttcacc-3′, respectively. The excised αv open reading frame was cloned into a pcDNA plasmid containing the sequence encoding the One Strep tag (GISGWSHPQFEKGGGSGSGGGSWSHPQFEK) in frame with the C-ter of αv-integrin.

### Antibodies and inhibitors

The M2 monoclonal antibody (MAb) anti-Flag was from Sigma-Aldrich; MAb anti-HA was from Covance. Polyclonal antibody (PAb) to gH/gL and MAb H170 to gD were described [53]. PAbs to Akt, phospho-Akt (Ser473), Src (36D10), phospho-Src (Tyr416), IRAK4, phospho-IRAK4 (Tyr345/Ser346) were from Cell Signaling. Strep-Tactin HRP (horseradish peroxidase conjugate) was from IBA GmbH (Gottinghen). PAb 1932 to β3-integrin was from Chemicon. The PP1 (4-Amino-5-(methylphenyl)-7-(t-butyl)pyrazolo-(3,4-d)pyrimidine) inhibitor of Src and the MK2206 inhibitor of Akt phosphorylation were from Sigma and Merck, respectively. MAb to IκB-α was from Cell Signaling and anti-tubulin MAb was from Sigma-Aldrich.

### NF-κB activity

293T or sh-β3 cells were transfected by means of Lipofectamine2000 (Invitrogen) with plasmid encoding firefly luciferase under a NF-κB regulated promoter, and Renilla luciferase in a 130∶1 NF-κB-luc∶Renilla ratio, plus TLR2-Flag or pcDNA 3.1 empty vector as indicated [8]. CD14, HA-PKB, PKB-CAAX, pCMV-HA-MYD88, pCMV-HA-MYD88_FW/AA_, β3_wt_, β3_Y747F_, β3_Y759F_ or β3_Y747-759F_ and αv-integrin, or a combination of plasmids, were included, as indicated in the text or figure legends. The transfected cells were maintained in pre-exhausted medium for two-three days prior to infection or LPS exposure [8]. Cells were exposed to 20 PFU/cell of the indicated virus for 6 h or LPS (Sigma-Aldrich, # L2630) (100 ng/ml) for 4 h. For treatment with inhibitors, cells were pre-treated for 1 h prior to exposure to virus (30 min for filipin III) and during virus absorption with the compounds. Luciferase activity was quantified by means of Dual Glo-luciferase reporter assay system (Promega).

#### Band assay

For detection and quantification of endogenous IκB-α, wt or sh β3 293T cells, transfected as indicated in the text or figure legends or treatment with inhibitors were infected with R7910 or gD^−/−^ virions. Lysate wewe harvested at 2 h after infection in buffer containing 20 mM Hepes, 250 mM NaCl, 1 mM EDTA, 1 mM DTT, 0.5% Igepal, plus protease and phosphatase inhibitors (Sigma), and separated by SDS PAGE after protein quantification (600 ng/lane). IκB-α, was detected by WB with anti.- IκB-α MAb. The blots were also reacted with anti-tubulin Mab (Sigma). To determine the amount of IFN-β wt or sh β3 293T cells were transfected as indicated in the text or figure legends or treatment with inhibitors. The cells culture media were harvested 48 h after infection with R7910 or gD^−/−^ virions and IFN-β was detected by means of VeriKine kit (Pestka Biomedical Laboratories, PBL INF Source).

Media were added to the pre-coated wells in a 1∶1 ratio with the kit dilution buffer for 2 h, according to the manufacturer's instructions. The bound IFN-β was revealed with antibody conjugated to peroxidase plus substrate and reading the optical density at 450 nm. Standard quantities of the purified IFN-β were run in parallel for relative quantification.

### Co-immunoprecipitation experiments

293T, HaCaT, HeLa or SK-N-SH cells, silenced or not for β3-integrin (cells were transfected by means of Lipofectamine2000 (Invitrogen) with TLR2-Flag-encoding plasmid (0.5 µg DNA for 10 cm^2^ dishes), plus plasmids encoding pCMV-HA-MYD88 (0.5 µg DNA for 10 cm^2^). When indicated sh-β3 cells were transfected with TRL2-Flag and pCMV-HA-MYD88 plasmids plus αv_wt_ plus β3_wt_ or αv_wt_ plus β3_Y747-759F_ for 48 h. 2–3 days after transfection, cells were exposed to R7910 (60 PFU/cell), or gH^−/−^ virions (60 PFU equivalent/cell), LPS (300 ng/ml) for 10, 20, 30 min at 37°C and lysed in PBS plus 1% DOC (deoxycholic acid), 1% Igepal containing the protease inhibitors *N* ^α^-*p*-tosyl-l-lysine chloromethyl ketone hydrochloride and *N* ^α^-*p*-tosyl-l-phenylalanine chloromethyl ketone (final concentration, 0.3 mM each), as detailed [9]. TLR2-Flag was immunoprecipitated with anti-Flag M2 MAb [9]. The proteins retained by Protein G-Sepharose, were separated by polyacrylamide gel electrophoresis (PAGE) and WB with MAb to HA or to Flag. To detect the interaction of TLR2 with gH/gL, 293T or 293T sh-β3 were transfected with plasmids encoding full length gH and gL plus TLR2-Flag; immunoprecipitation was performed as described above by means of anti-Flag MAb; gH was detected by means of PAb to gH/gL. To detect the TLR2 and β3-integrin interaction, sh-β3 cells were transfected with αv_wt_ plus β3_wt_ or αv_wt_ plus β3_Y747-759F_ and TLR2-Flag. TLR2 was immonoprecipitated by means of anti-Flag MAb. β3-integrin was detected by WB with PAb 1932.

To determine IRAK4 phosphorylation, 293T or sh-β3 cells were transfected with TLR2-Flag-encoding plasmid, plus plasmids encoding HA-MYD88. Cells were exposed to R7910 or LPS (300 ng/ml), as detailed above, and lysed in RIPA buffer (20 mM HEPES (4-(2-hydroxyethyl)-1-piperazineethanesulfonic acid), 250 mM NaCl, 1 mM EDTA, 1 mM DTT, 0.5% Igepal) containing the protease inhibitors and the phosphatase inhibitors cocktail (Sigma-Aldrich). The endogenous IRAK4 was immunoprecipitated with PAb to IRAK4; phosphorylation was detected by means of anti phospho-IRAK4 PAb. The αv-integrin precipitation was carried out from sh-β3 cells, previously transfected with αv_st_ plus β3_wt_ or αv_st_ plus β3_Y747-759F_, plus pCMV-HA-MYD88 plasmids, for 48 h; when indicated, TLR2-Flag was included. Following infection with R7910, the cells were lysed in EA1 buffer plus (50 mM HEPES, 250 mM NaCl, 0.5% Igepal, pH 8) containing 0.3 mM protease inhibitors. αv_st_ was harvested with Strep-Tactin Sepharose (IBA, GmbH, Gottingen, Germany) [53]; the retained proteins were separated by PAGE and blotted with MAb to HA to detect MYD88, or to FLAG to detect TLR2.

### Membrane flotation in sucrose gradients

293T or 293T sh-β3 cells were transfected with plasmids encoding TLR2-Flag, plus pCMV-HA-MYD88 and pEFBos MAL Flag. When indicated, CD14 was included. 48 h after transfection, the cells were exposed for 30 min to R7910 (60 PFU/cell) at 37°C or mock-infected. Cells were harvested, suspended in 1 ml of TNE buffer (10 mM Tris-HCl, pH 7.5, 150 mM NaCl, 5 mM EDTA) containing 1% Triton X-100 (Sigma Aldrich, Milan, Italy) and 0.3 mM protease inhibitors, and incubated on ice for 1 h. Membrane fractions were prepared essentially as described [26]. The samples obtained from fractionation of the sucrose gradient were subjected to PAGE and blotted with MAbs to HA (for detection of MYD88) and to Flag (for detection of TLR2 and MAL).

### Protein phosphorylation

293T HaCaT, HeLa or SK-N-SH cells, silenced or not for β3-integrin were transfected or not with TLR2-Flag encoding plasmid. 293T sh-β3 cells were transfected with αv-integrin plasmid plus β3_wt_ or β3_Y747-759F_ and exposed to R7910 (60 PFU/cell) or, gH^−/−^ virions (60 PFU equivalent/cell for 10, 20 and 30 min at 37°C. Cells were lysed with RIPA buffer. 300 ng of total proteins were subjected to PAGE. Src, phospho-Src, Akt and phospho-Akt were detected by WB with appropriate antibodies in two separate gels (one for Src and one for Akt generated in parallel in the same experiment).

### R7910 yield

293T or 293T sh-β3 cells were transfected with plasmids encoding TLR2-Flag plus pCMV-HA-MYD88, pCMV-HA-MYD88_FW/AA_, HA-PKB or PKB-CAAX. 24 h after transfection cells were infected with R7910 (1 PFU/cell) for 90 min at 37°C. Extracellular virus was inactivated by means of an acidic wash (40 mM citric acid, 10 mM KCl, 135 mM NACl, pH 3). Replicate cultures were frozen at 3, 24 or 48 h after infection and viral progeny (intracellular plus extracellular) was titrated on U20S cells.

## References

[1] Kawai T, Akira S (2010) The role of pattern-recognition receptors in innate immunity: update on Toll-like receptors. Nat Immunol 11: 373–384. 20404851 10.1038/ni.1863

[2] Rathinam VA, Fitzgerald KA (2011) Innate immune sensing of DNA viruses. Virology 411: 153–162. 21334037 10.1016/j.virol.2011.02.003 PMC3070751

[3] Barbalat R, Lau L, Locksley RM, Barton GM (2009) Toll-like receptor 2 on inflammatory monocytes induces type I interferon in response to viral but not bacterial ligands. Nat Immunol 10: 1200–1207. 19801985 10.1038/ni.1792 PMC2821672

[4] Finberg RW, Knipe DM, Kurt-Jones EA (2005) Herpes simplex virus and toll-like receptors. Viral Immunol 18: 457–465. 16212524 10.1089/vim.2005.18.457

[5] van Bergenhenegouwen J, Plantinga TS, Joosten LA, Netea MG, Folkerts G, et al (2013) TLR2 & Co: a critical analysis of the complex interactions between TLR2 and coreceptors. J Leukoc Biol 94: 885–902. 23990624 10.1189/jlb.0113003

[6] Gerold G, Ajaj KA, Bienert M, Laws HJ, Zychlinsky A, et al (2008) A Toll-like receptor 2-integrin beta3 complex senses bacterial lipopeptides via vitronectin. Nat Immunol 9: 761–768. 18516040 10.1038/ni.1618

[7] Gianni T, Leoni V, Campadelli-Fiume G (2013) Type I interferon and NF-kappaB activation elicited by herpes simplex virus gH/gL via alphavbeta3 integrin in epithelial and neuronal cell lines. J Virol 87: 13911–13916. 24109241 10.1128/JVI.01894-13 PMC3838217

[8] Gianni T, Leoni V, Chesnokova LS, Hutt-Fletcher LM, Campadelli-Fiume G (2012) alphavbeta3-integrin is a major sensor and activator of innate immunity to herpes simplex virus-1. Proc Natl Acad Sci U S A 109: 19792–19797. 23150579 10.1073/pnas.1212597109 PMC3511702

[9] Leoni V, Gianni T, Salvioli S, Campadelli-Fiume G (2012) Herpes Simplex Virus Glycoproteins gH/gL and gB Bind Toll-Like Receptor 2, and Soluble gH/gL Is Sufficient To Activate NF-kappaB. J Virol 86: 6555–6562. 22496225 10.1128/JVI.00295-12 PMC3393584

[10] Paludan SR, Bowie AG, Horan KA, Fitzgerald KA (2011) Recognition of herpesviruses by the innate immune system. Nat Rev Immunol 11: 143–154. 21267015 10.1038/nri2937 PMC3686362

[11] Paladino P, Mossman KL (2009) Mechanisms employed by herpes simplex virus 1 to inhibit the interferon response. J Interferon Cytokine Res 29: 599–607. 19694546 10.1089/jir.2009.0074

[12] Unterholzner L (2013) The interferon response to intracellular DNA: why so many receptors? Immunobiology 218: 1312–1321. 23962476 10.1016/j.imbio.2013.07.007

[13] Kalamvoki M, Roizman B (2014) HSV-1 degrades, stabilizes, requires, or is stung by STING depending on ICP0, the US3 protein kinase, and cell derivation. Proc Natl Acad Sci U S A 111: E611–617. 24449861 10.1073/pnas.1323414111 PMC3918790

[14] Campadelli-Fiume G, Menotti L, Avitabile E, Gianni T (2012) Viral and cellular contributions to herpes simplex virus entry into the cell. Curr Opin Virol 2: 28–36. 22440963 10.1016/j.coviro.2011.12.001

[15] Connolly SA, Jackson JO, Jardetzky TS, Longnecker R (2011) Fusing structure and function: a structural view of the herpesvirus entry machinery. Nat Rev Microbiol 9: 369–381. 21478902 10.1038/nrmicro2548 PMC3242325

[16] Roizman B (2011) The checkpoints of viral gene expression in productive and latent infection: the role of the HDAC/CoREST/LSD1/REST repressor complex. J Virol 85: 7474–7482. 21450817 10.1128/JVI.00180-11 PMC3147896

[17] Gao C, Schaefer E, Lakkis M, Blystone SD (2005) Beta3 tyrosine phosphorylation and alphavbeta3-mediated adhesion are required for Vav1 association and Rho activation in leukocytes. J Biol Chem 280: 15422–15429. 15699036 10.1074/jbc.M414457200

[18] Lopez P, Jacob RJ, Roizman B (2002) Overexpression of promyelocytic leukemia protein precludes the dispersal of ND10 structures and has no effect on accumulation of infectious herpes simplex virus 1 or its proteins. J Virol 76: 9355–9367. 12186918 10.1128/JVI.76.18.9355-9367.2002 PMC136451

[19] Kawai T, Akira S (2011) Toll-like receptors and their crosstalk with other innate receptors in infection and immunity. Immunity 34: 637–650. 21616434 10.1016/j.immuni.2011.05.006

[20] Jiang Z, Georgel P, Li C, Choe J, Crozat K, et al (2006) Details of Toll-like receptor:adapter interaction revealed by germ-line mutagenesis. Proc Natl Acad Sci U S A 103: 10961–10966. 16832055 10.1073/pnas.0603804103 PMC1544157

[21] Cheshenko N, Trepanier JB, Stefanidou M, Buckley N, Gonzalez P, et al (2013) HSV activates Akt to trigger calcium release and promote viral entry: novel candidate target for treatment and suppression. FASEB J 27: 2584–2599. 23507869 10.1096/fj.12-220285 PMC3688744

[22] Benetti L, Roizman B (2006) Protein kinase B/Akt is present in activated form throughout the entire replicative cycle of deltaU(S)3 mutant virus but only at early times after infection with wild-type herpes simplex virus 1. J Virol 80: 3341–3348. 16537601 10.1128/JVI.80.7.3341-3348.2006 PMC1440418

[23] van Weeren PC, de Bruyn KM, de Vries-Smits AM, van Lint J, Burgering BM (1998) Essential role for protein kinase B (PKB) in insulin-induced glycogen synthase kinase 3 inactivation. Characterization of dominant-negative mutant of PKB. J Biol Chem 273: 13150–13156. 9582355 10.1074/jbc.273.21.13150

[24] Forrester A, Farrell H, Wilkinson G, Kaye J, Davis Poynter N, et al (1992) Construction and properties of a mutant of herpes simplex virus type 1 with glycoprotein H coding sequences deleted. J Virol 66: 341–348. 1309250 10.1128/jvi.66.1.341-348.1992 PMC238293

[25] Jiang Z, Georgel P, Du X, Shamel L, Sovath S, et al (2005) CD14 is required for MyD88-independent LPS signaling. Nat Immunol 6: 565–570. 15895089 10.1038/ni1207

[26] Gianni T, Campadelli-Fiume G, Menotti L (2004) Entry of Herpes Simplex Virus Mediated by Chimeric Forms of Nectin1 Retargeted to Endosomes or to Lipid Rafts Occurs through Acidic Endosomes. J Virol 78: 12268–12276. 15507614 10.1128/JVI.78.22.12268-12276.2004 PMC525084

[27] Gianni T, Salvioli S, Chesnokova LS, Hutt-Fletcher LM, Campadelli-Fiume G (2013) alphavbeta6- and alphavbeta8-integrins serve as interchangeable receptors for HSV gH/gL to promote endocytosis and activation of membrane fusion. PLoS Pathog 9: e1003806. 24367260 10.1371/journal.ppat.1003806 PMC3868510

[28] Holm CK, Jensen SB, Jakobsen MR, Cheshenko N, Horan KA, et al (2012) Virus-cell fusion as a trigger of innate immunity dependent on the adaptor STING. Nat Immunol 10.1038/ni.2350 PMC3411909 22706339

[29] Medici MA, Sciortino MT, Perri D, Amici C, Avitabile E, et al (2003) Protection by herpes simplex virus glycoprotein D against Fas-mediated apoptosis: role of nuclear factor kappaB. J Biol Chem 278: 36059–36067. 12844494 10.1074/jbc.M306198200

[30] Paladino P, Cummings DT, Noyce RS, Mossman KL (2006) The IFN-independent response to virus particle entry provides a first line of antiviral defense that is independent of TLRs and retinoic acid-inducible gene I. J Immunol 177: 8008–8016. 17114474 10.4049/jimmunol.177.11.8008

[31] Cheshenko N, Liu W, Satlin LM, Herold BC (2005) Focal adhesion kinase plays a pivotal role in herpes simplex virus entry. J Biol Chem 280: 31116–31125. 15994312 10.1074/jbc.M503518200

[32] Gregory D, Hargett D, Holmes D, Money E, Bachenheimer SL (2004) Efficient replication by herpes simplex virus type 1 involves activation of the IkappaB kinase-IkappaB-p65 pathway. J Virol 78: 13582–13590. 15564469 10.1128/JVI.78.24.13582-13590.2004 PMC533927

[33] Roberts KL, Baines JD (2011) UL31 of herpes simplex virus 1 is necessary for optimal NF-kappaB activation and expression of viral gene products. J Virol 85: 4947–4953. 21389131 10.1128/JVI.00068-11 PMC3126170

[34] Taddeo B, Luo TR, Zhang W, Roizman B (2003) Activation of NF-kappaB in cells productively infected with HSV-1 depends on activated protein kinase R and plays no apparent role in blocking apoptosis. Proc Natl Acad Sci U S A 100: 12408–12413. 14530405 10.1073/pnas.2034952100 PMC218771

[35] Chuluunbaatar U, Roller R, Feldman ME, Brown S, Shokat KM, et al (2010) Constitutive mTORC1 activation by a herpesvirus Akt surrogate stimulates mRNA translation and viral replication. Genes Dev 24: 2627–2639. 21123650 10.1101/gad.1978310 PMC2994037

[36] Eaton HE, Saffran HA, Wu FW, Quach K, Smiley JR (2014) Herpes Simplex Virus Protein Kinases US3 and UL13 Modulate VP11/12 Phosphorylation, Virion Packaging, and Phosphatidylinositol 3-Kinase/Akt Signaling Activity. J Virol 88: 7379–7388. 24741093 10.1128/JVI.00712-14 PMC4054420

[37] Sen J, Liu X, Roller R, Knipe DM (2013) Herpes simplex virus US3 tegument protein inhibits Toll-like receptor 2 signaling at or before TRAF6 ubiquitination. Virology 439: 65–73. 23478027 10.1016/j.virol.2013.01.026 PMC3810314

[38] van Lint AL, Murawski MR, Goodbody RE, Severa M, Fitzgerald KA, et al (2010) Herpes simplex virus immediate-early ICP0 protein inhibits Toll-like receptor 2-dependent inflammatory responses and NF-kappaB signaling. J Virol 84: 10802–10811. 20686034 10.1128/JVI.00063-10 PMC2950559

[39] Lowell CA (2011) Src-family and Syk kinases in activating and inhibitory pathways in innate immune cells: signaling cross talk. Cold Spring Harb Perspect Biol 3. 10.1101/cshperspect.a002352 PMC3039931 21068150

[40] Cheshenko N, Trepanier JB, Gonzalez PA, Eugenin EA, Jacobs WR Jr, et al (2014) Herpes Simplex Virus Type 2 Glycoprotein H Interacts with Integrin alphavbeta3 To Facilitate Viral Entry and Calcium Signaling in Human Genital Tract Epithelial Cells. J Virol 88: 10026–10038. 24942591 10.1128/JVI.00725-14 PMC4136333

[41] Moore KJ, Andersson LP, Ingalls RR, Monks BG, Li R, et al (2000) Divergent response to LPS and bacteria in CD14-deficient murine macrophages. J Immunol 165: 4272–4280. 11035061 10.4049/jimmunol.165.8.4272

[42] Gianni T, Campadelli-Fiume G (2012) alphaVbeta3-integrin relocalizes nectin1 and routes herpes simplex virus to lipid rafts. J Virol 86: 2850–2855. 22171266 10.1128/JVI.06689-11 PMC3302258

[43] Triantafilou M, Gamper FG, Haston RM, Mouratis MA, Morath S, et al (2006) Membrane sorting of toll-like receptor (TLR)-2/6 and TLR2/1 heterodimers at the cell surface determines heterotypic associations with CD36 and intracellular targeting. J Biol Chem 281: 31002–31011. 16880211 10.1074/jbc.M602794200

[44] Schmitz G, Orso E (2002) CD14 signalling in lipid rafts: new ligands and co-receptors. Curr Opin Lipidol 13: 513–521. 12352015 10.1097/00041433-200210000-00007

[45] Heit B, Kim H, Cosio G, Castano D, Collins R, et al (2013) Multimolecular signaling complexes enable Syk-mediated signaling of CD36 internalization. Dev Cell 24: 372–383. 23395392 10.1016/j.devcel.2013.01.007 PMC3586299

[46] Abe T, Shimamura M, Jackman K, Kurinami H, Anrather J, et al (2010) Key role of CD36 in Toll-like receptor 2 signaling in cerebral ischemia. Stroke 41: 898–904. 20360550 10.1161/STROKEAHA.109.572552 PMC2950279

[47] Hale JS, Otvos B, Sinyuk M, Alvarado AG, Hitomi M, et al (2014) Cancer stem cell-specific scavenger receptor CD36 drives glioblastoma progression. Stem Cells 10.1002/stem.1716 PMC4063873 24737733

[48] Marre ML, Petnicki-Ocwieja T, DeFrancesco AS, Darcy CT, Hu LT (2010) Human integrin alpha(3)beta(1) regulates TLR2 recognition of lipopeptides from endosomal compartments. PLoS One 5: e12871. 20877569 10.1371/journal.pone.0012871 PMC2943923

[49] Kagan JC, Medzhitov R (2006) Phosphoinositide-mediated adaptor recruitment controls Toll-like receptor signaling. Cell 125: 943–955. 16751103 10.1016/j.cell.2006.03.047

[50] Ling GS, Bennett J, Woollard KJ, Szajna M, Fossati-Jimack L, et al (2014) Integrin CD11b positively regulates TLR4-induced signalling pathways in dendritic cells but not in macrophages. Nat Commun 5: 3039. 24423728 10.1038/ncomms4039 PMC3905776

[51] Rauch DA, Rodriguez N, Roller RJ (2000) Mutations in herpes simplex virus glycoprotein D distinguish entry of free virus from cell-cell spread. J Virol 74: 11437–11446. 11090139 10.1128/jvi.74.24.11437-11446.2000 PMC112422

[52] Avitabile E, Lombardi G, Gianni T, Capri M, Campadelli-Fiume G (2004) Coexpression of UL20p and gK inhibits cell-cell fusion mediated by herpes simplex virus glycoproteins gD, gH-gL, and wt- gB or an endocytosis-defective gB mutant, and downmodulates their cell surface expression. J Virol 78: 8015–8025. 15254173 10.1128/JVI.78.15.8015-8025.2004 PMC446093

[53] Gianni T, Cerretani A, Dubois R, Salvioli S, Blystone SS, et al (2010) Herpes simplex virus glycoproteins H/L bind to cells independently of {alpha}V{beta}3 integrin and inhibit virus entry, and their constitutive expression restricts infection. J Virol 84: 4013–4025. 20147400 10.1128/JVI.02502-09 PMC2849490

